# Pharmacological blocking of neutrophil extracellular traps attenuates immunothrombosis and neuroinflammation in cerebral cavernous malformation

**DOI:** 10.1038/s44161-024-00577-y

**Published:** 2024-12-04

**Authors:** Favour C. Onyeogaziri, Ross Smith, Maximiliano Arce, Hua Huang, Iza Erzar, Charlotte Rorsman, Matteo Malinverno, Fabrizio Orsenigo, Veronica Sundell, Dinesh Fernando, Geoffrey Daniel, Mika Niemelä, Aki Laakso, Behnam Rezai Jahromi, Anna-Karin Olsson, Peetra U. Magnusson

**Affiliations:** 1 Department of Immunology, Genetics and Pathology, Uppsala University, Uppsala, Sweden; 2 Vascular Biology Unit, The FIRC Institute of Molecular Oncology Foundation, Milan, Italy; 3 Department of Biomaterials and Technology/Wood Science, Swedish University of Agricultural Sciences, Uppsala, Sweden; 4 Department of Neurosurgery, University of Helsinki and Helsinki University Hospital, Helsinki, Finland; 5 Department of Medical Biochemistry and Microbiology, Uppsala University, Uppsala, Sweden

**Keywords:** Inflammation, Coagulation system

## Abstract

Cerebral cavernous malformation (CCM) is a neurovascular disease with symptoms such as strokes, hemorrhages and neurological deficits. With surgery being the only treatment strategy, understanding the molecular mechanisms of CCM is crucial in finding alternative therapeutic options for CCM. Neutrophil extracellular traps (NETs) were recently reported in CCM, and NETs were shown to have positive or negative effects in different disease contexts. In this study, we investigated the roles of NETs in CCM by pharmacologically inhibiting NET formation using Cl-amidine (a peptidyl arginine deiminase inhibitor). We show here that Cl-amidine treatment reduced lesion burden, coagulation and endothelial-to-mesenchymal transition. Furthermore, NETs promoted the activation of microglia and fibroblasts, leading to increased neuroinflammation and a chronic wound microenvironment in CCM. The inhibition of NET formation caused endothelial quiescence and promoted a healthier microenvironment. Our study suggests the inhibition of NETs as a potential therapeutic strategy in CCM.

## Abstract (web summary)

Onyeogaziri et al. show that the formation of neutrophil extracellular traps contributes to a chronic wound state in cerebral cavernous malformation, while inhibition of these traps with CI-amidine establishes a healthier microenvironment and promotes endothelial cell quiescence, suggesting use of CI-amidine as a potential therapeutic strategy.

## Main

Cerebral cavernous malformation (CCM) is a disease of the central nervous system marked by hemorrhages, strokes, seizures and other neurological issues, with surgery being the only treatment option^1^. CCM exists as a sporadic or familial autosomal dominant disease, with both forms being linked to loss-of-function mutations in CCM1/*KRIT1*, CCM2/malcavernin and CCM3/*PDCD10* (refs. ^2,3^). Recent findings have also linked gain-of-function mutations in the *PIK3CA* gene to both forms of CCM^4,5^. Although the mechanisms that drive the initiation and progression of CCM are not fully understood, disrupted cell–cell junctions, increased vascular permeability, endothelial-to-mesenchymal transition (EndMT), clonal expansion, altered basement membrane (BM) composition, inflammation and dysregulated hemostasis were shown to be involved in CCM pathogenesis^6–12^.

Although it is well established that CCM is a disease of the endothelium, the involvement of non-endothelial cells is now being uncovered^13–18^. In particular, immune cells are at the center of multiple pathways dysregulated in CCM, such as coagulation, reactive oxygen species formation and hypoxia^19–21^. The involvement of inflammatory cells in CCM is recognized^22,23^, but the roles of neutrophils, which are the most abundant leukocytes in blood, have not been well studied in CCM. We recently showed that in CCM, neutrophils become activated and are able to extrude neutrophil extracellular traps (NETs) in a process called NETosis/NET formation^24^.

NETosis is a host response carried out by neutrophils to remove pathogens, involving neutrophil lysis and the extrusion of their DNA^25^. Described in brief, neutrophil elastase (NE) and myeloperoxidase (MPO) from cytoplasmic granules translocate to the nucleus and promote the decondensation of nuclear chromatins and activation of peptidyl arginine deiminase 4 (PAD4). PAD4 then citrullinates histones, particularly histone 3 (citH3), allowing complete chromatin decondensation. After nuclear breakdown, the decondensed chromatin enters the cytosol where it becomes coated with other granular proteins and is released from the neutrophil^26^. Although NETosis is a physiological response, chronic and continuous production of NETs, as seen in sepsis, cancer, coronavirus disease 2019 (COVID-19) and autoimmune diseases, is harmful^27–29^. This is because products released during NET formation, such as MPO, NE and cathepsin G, have a broad range of target molecules and can cause damage to cells, recruit other inflammatory cells or induce the production of autoantibodies^25,30^.

As previously reported, short-term DNase-I-mediated degradation of NETs in a CCM murine model partially rescued the vascular barrier^24^, suggesting that NETs are detrimental in CCM and might represent a potential therapeutic avenue. However, the role of NET formation in CCM pathogenesis is still unclear. In the present study, we aimed to further understand the role of NETs in CCM via the inhibition of PAD4 using *N*-α-benzoyl-*N5*-(2-chloro-1-iminoethyl)-l-ornithine amide (Cl-amidine). The pan-PAD irreversible inhibitor Cl-amidine was shown to be highly effective for inhibiting PAD4 (ref. ^31^), with high specificity for PAD4 and preferential inhibition over PAD2 (ref. ^32^). Furthermore, Cl-amidine was shown to improve inflammatory-related diseases in murine models^33–35^. In this study, using a murine model of endothelial-specific deletion of *Ccm3*, we reveal that Cl-amidine decreased NET formation, resulting in reduced lesion burden, endothelial activation, EndMT and coagulation as well as a reduction of activated fibroblasts and microglial cells. Furthermore, we report and characterize the presence of organized clots in CCM. These are chronic clots that become reorganized in a process similar to wound healing, leading to increased presence of leukocytes, collagen deposition and fibrosis, which ultimately reduce vessel perfusion and clot lysis^36^.

## Results

### Cl-amidine reduces NET formation and lesion burden in CCM

**Fig. 1:**
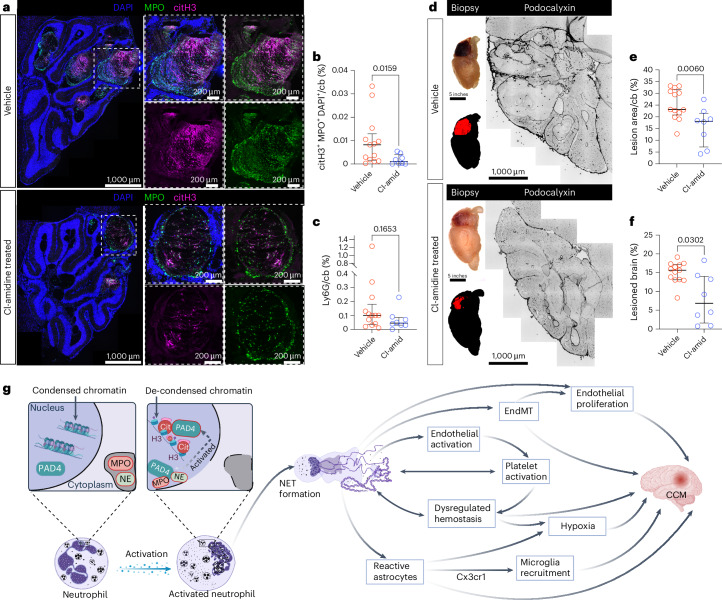
Cl-amidine treatment attenuated NETs and lesion formation in CCM. **a**, Representative images of the cerebellum of *Ccm3-iECKO* vehicle-treated mice (upper panel) and Cl-amidine-treated (10 mg kg^−1^ d^−1^) mice (lower panel) stained with DAPI (blue), MPO (green) and citH3 (magenta). Insets (right) are zoomed-in regions. **b**,**c**, Quantification of citH3^+^MPO^+^DAPI^+^ NETs (**b**) and Ly6G (**c**) in the cerebellum of *Ccm3-iECKO* vehicle-treated (*n* = 13) and Cl-amidine-treated (10 mg kg^−1^ d^−1^; *n* = 8) mice. **d**, Representative images of mouse brains used in the study and the output from the semi-automated quantification of lesion burden in the brain of *Ccm3-iECKO* vehicle-treated and Cl-amidine-treated (10 mg kg^−1^ d^−1^) mice. **e**,**f**, Quantification of lesion area (**e**) and lesion burden (**f**) within the cerebellum of *Ccm3-iECKO* vehicle-treated (*n* = 13) and Cl-amidine-treated (10 mg kg^−1^ d^−1^; *n* = 8) mice. In the graphs, each data point represents one biological replicate; the bar indicates the median of each group; and the error bars represent the IQR. Statistical significance was determined using a Mann–Whitney *U*-test (two-tailed). **g**, Schematic depicting the process of NET formation (left) and the pathways affected by NETs and CCM. NETs were reported to induce endothelial activation^43^. Endothelial activation facilitates the binding of platelets^42,47^, leading to increased thrombi formation. NETs can also directly induce platelet activation^27^. Continuous thrombi formation would lead to ischemia/hypoxia within the brain. EndMT is a known driver of CCM lesion progression^46^ and was reported to be promoted by NETosis^45^. Endothelial cells that undergo EndMT become mesenchymal and, consequently, more proliferative and highly mobile^46^, leading to lesion growth. Reactive astrogliosis was shown to promote CCM pathogenesis directly through HIF1/VEGF hypoxia pathway^10^ and indirectly by attracting microglial cells via CX3CR1 (ref. ^14^). This figure was created with BioRender. cb, cerebellum. Source data

**Extended Data Fig. 1:**
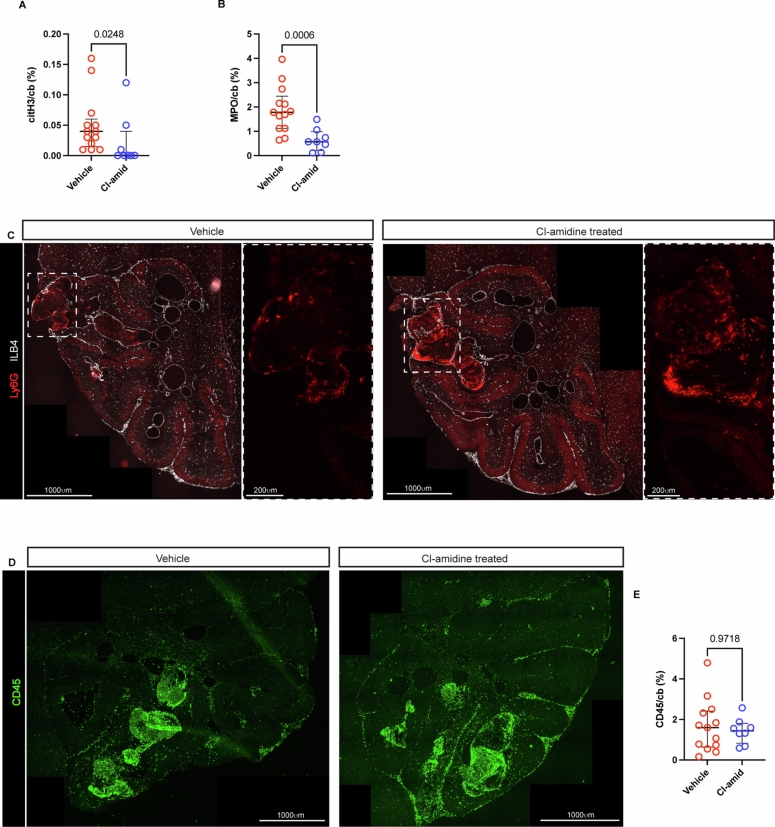
Cl-amidine’s effect on immune cells. (**a, b**) Quantification of citH3^+^ (**a**), MPO^+^ (**b**) in the cerebellum (cb) of *Ccm3-iECKO* vehicle and Cl-amidine (10 mg/kg/day) treated mice. (**c**) Representative images of the cerebellum of *Ccm3-iECKO* vehicle and Cl-amidine treated mice stained with Ly6G (red), and ILB4 (white). (**d**) Representative images of the cerebellum of *Ccm3-iECKO* vehicle and Cl-amidine treated mice stained with CD45 (green) to detect immune cells. (**e**) Quantification of CD45 in the cerebellum (cb) of *Ccm3-iECKO* vehicle (n = 13) and Cl-amidine (10 mg/kg/day; n = 8) treated mice. In the graphs, each data point represents one biological replicate, the bar indicates the median of each group, and the error bars represent the interquartile range. Statistical significance was determined using a Mann–Whitney U test (two tailed). Source data

The ability of Cl-amidine to inhibit PAD4 and, consequently, NET formation has been well described^32,34^. To ascertain that the established dose of 10 mg kg^−1^ (refs. ^32,34^) was therapeutically sufficient in CCM, we analyzed the NET burden in the treated mice compared to controls. Using immunofluorescence, NETs were defined as the regions triple positive for citH3, MPO and DAPI. We observed an 87.5% significant decrease of citH3^+^/MPO^+^/DAPI^+^ area in the Cl-amidine-treated mice (*P* = 0.0159) (Fig. 1a,b). The expression of citH3 and MPO, measured individually, was also significantly reduced in the Cl-amidine-treated group compared to vehicle controls (*P* = 0.0248 and *P* = 0.0006, respectively; Extended Data Fig. 1a,b). To confirm that the reduction in NETs was not simply because of reduced numbers of neutrophils upon Cl-amidine treatment, the expression of Ly6G was assayed via immunofluorescence staining. No difference was observed in the Ly6G expression between the two groups (*P* = 0.1653) (Fig. 1c and Extended Data Fig. 1c). There was also no difference in the CD45^+^ immune cells between the groups (*P* = 0.9718) (Extended Data Fig. 1d,e). This is line with previous reports that the effect of Cl-amidine is limited to neutrophils and their capability of producing NETs^32,34^.

**Extended Data Fig. 2:**
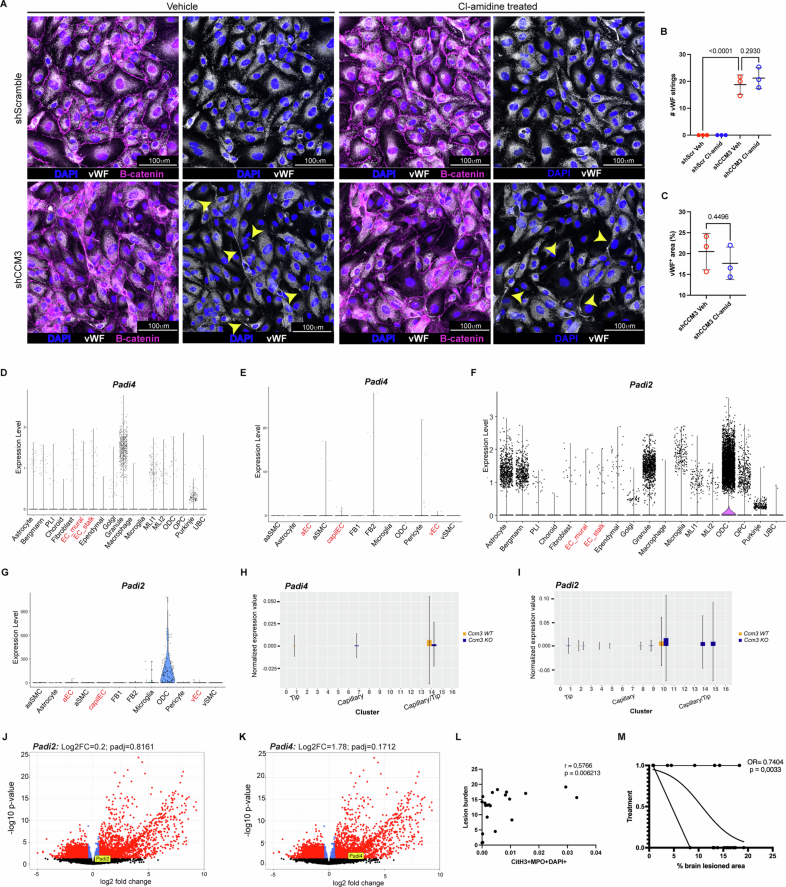
*Padi2* and *Padi4* expression in mouse brain is minimal. (**a**) Representative images of HBMVECs (shScramble, top and *shCCM3*, bottom) treated with vehicle control (left) or 6 μM Cl-amidine (right) for 24 h. Stained for vWF (white), B-catenin (magenta), and DAPI (blue). Arrows highlight vWF strings. (**b**) Quantification of the number of vWF strings per field of view in shScramble (shScr) and shCCM3 HBMVECs treated with vehicle or 6 μM Cl-amidine. Each data point represents 1 biological replicate (n = 3) with three fields of view imaged and quantified per condition. The bar indicates the mean of each group, and the error bars represent the standard deviation. Statistical significance was determined using one-way ANOVA followed by a post-hoc Holm-sidak’s test. (**c**) Quantification of the number of total vWF area per field of view in shCCM*3* HBMVECs treated with vehicle or 6 μM Cl-amidine. Each data point represents 1 biological replicate (n = 3) with three fields of view imaged per condition. The bar indicates the mean of each group, and the error bars represent the standard deviation. (**d, e**) Gene expression of *Padi4* in mouse brain cerebellum (**D**) and vascular and perivascular cells (**E**). (**f, g**) Gene expression of *Padi2* in mouse brain cerebellum (**F**) and vascular and perivascular cells (**G**). (**h, i**): Bulk RNA-seq data of *Padi4* (**H**) and *Padi2* (**I**) in mouse brain endothelial cells. The error bars represent the standard deviation. (**J**-**K**) single cell RNA-seq data of *Padi4* (**j**) and *Padi2* (**k**) in mouse brain cells; Thresholds: blue signifies log2FC ≥ 0.5 & padj ≤ 0.05, red signifies log2FC < 0.5 & padj ≤ 0.05, black signifies p value > 0.05. (**l**) Correlation analysis of lesion burden to NET burden characterized by citH3+MPO + DAPI+ signals. (**m**) Logistic regression graph showing the relationship between receiving treatment (1) or not (0) and lesion burden. OR: odds ratio, p: significance level. Source data

Cl-amidine will attenuate the activity of PADs irrespective of what cell type expresses it. Citrullination of proteins other than histones has been reported. In mesenteric vessels, citrullination of ADAMTS13 inhibits its ability to cleave von Willebrand factor (vWF) strings^37^. To ascertain if Cl-amidine had a direct effect on brain endothelial cell–specific PADs, shScramble and shCCM3 brain endothelial cells (human brain microvascular endothelial cells (HBMVECs)) were treated with vehicle or 6 μM Cl-amidine. vWF strings are released in HBMVECs after loss of CCM3 (ref. ^8^); thus, the presence of vWF strings was quantified as the readout of PAD inhibition. No difference was observed in the number of vWF strings (*P* = 0.2930) or total vWF expression (*P* = 0.4496) in the shCCM3 cells treated with Cl-amidine compared to controls (Extended Data Fig. 2a–c). This suggests that citrullination was not increased in brain endothelial cells after loss of CCM3.

To investigate the expression of *Padi4* and *Padi2* (genes encoding PAD4 and PAD2) in the mouse brain, publicly available datasets from mouse cerebellar single-nucleus RNA sequencing (RNA-seq)^38^ and mouse vascular/perivascular RNA-seq^39,40^ were analyzed. We found that *Padi4* and *Padi2* expression in brain cells is minimal (Extended Data Fig. 2d–g). To exclude the possibility that the loss of CCM3 in endothelial cells results in PAD4 and PAD2 upregulation, we analyzed our previously published bulk (Gene Expression Omnibus (GEO): GSE246373)^24^ and single-cell RNA-seq (GEO: GSE155788)^41^ data to investigate the expression of *Padi4* and *Padi2* after CCM3 loss. Our analysis revealed that *Padi4* and *Padi2* expression were not affected by loss of CCM3 (Extended Data Fig. 2h–k); therefore, Cl-amidine should not have any direct effects on the endothelial cells related to specific PAD inhibition. In concert, these data indicate that any effects observed after Cl-amidine treatment in mice are primarily due to its inhibition of neutrophil PAD4 and, consequently, NET formation.

To assess the relationship between the presence of NETs and lesion burden, a correlation test was done. It showed a positive, moderate correlation between lesion burden and NET burden (*r* = 0.5766; *P* = 0.006213) (Extended Data Fig. 2l). This suggests that the higher the NET burden, the higher the lesion burden. To elucidate if the formation of NETs plays a role in CCM lesion formation or progression, the lesion burden was assessed after Cl-amidine treatment. Lesion formation is restricted mainly to the cerebellum for the mouse model used in this study. Thus, manual counting of the lesions within the cerebellum was carried out. This showed that there was a significant decrease in cerebellar lesion area in the Cl-amidine-treated group compared to vehicle (Fig. 1d,e; *P* = 0.0060). To further confirm this, the overall lesion burden was quantified using an additional semi-automated lesion detection method, wherein we also detected a significant reduction (56.3%) in the lesion burden of Cl-amidine-treated mice (*P* = 0.0302) (Fig. 1d,f). Logistic regression analysis of the data showed an odds ratio of 0.7404 (*P* = 0.0033), indicating a 26% decrease in risk of brain lesions after Cl-amidine treatment (Extended Data Fig. 2m).

To understand how inhibiting NET formation could affect lesion progression, pathways previously reported to be involved in CCM pathogenesis and linked to NETs^14,27,42–47^ were further studied (see schematic in Fig. 1g).

### Cl-amidine decreases endothelial dysfunction in CCM

**Fig. 2:**
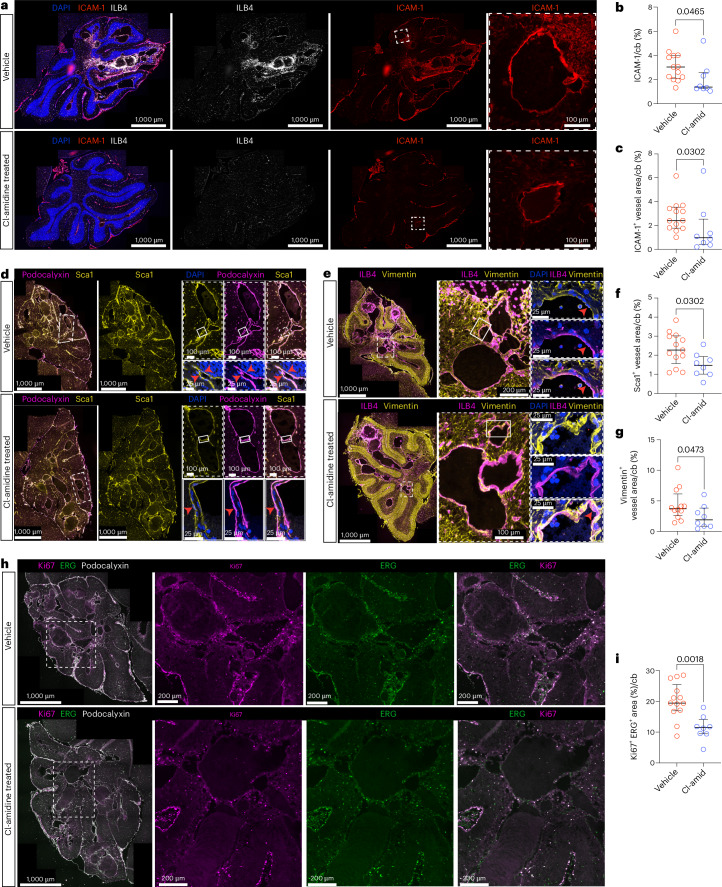
Cl-amidine reduced endothelial activation, EndMT and proliferation in CCM. **a**, Representative images of the cerebellum of *Ccm3-iECKO* vehicle-treated mice (upper panel) and Cl-amidine-treated (10 mg kg^−1^ d^−1^) mice (lower panel) stained with DAPI (blue), ICAM-1 (red) and ILB4 (white). Insets with white line highlight magnifications to the right. **b**,**c**, Quantification of total ICAM-1^+^ area (**b**) and vascular (ILB4^+^) ICAM-1^+^ area (**c**) in the cerebellum of *Ccm3-iECKO* vehicle-treated (*n* = 13) and Cl-amidine-treated (10 mg kg^−1^ d^−1^; *n* = 8) mice. **d**, Representative images of endothelial cells undergoing EndMT in the cerebellum of *Ccm3-iECKO* mice stained with DAPI (blue), Sca1 (yellow) and podocalyxin (magenta). Co-localization of Sca1 and podocalyxin is shown as white. Insets with dashed, white line are shown in the magnifications to the right. Insets with white line are shown in the magnifications to the bottom, showing zoomed-in areas with EndMT with nuclei stained with DAPI (blue). **e**, Representative images of endothelial cells undergoing EndMT in the cerebellum of *Ccm3-iECKO* mice stained with DAPI (blue), vimentin (yellow) and ILB4 (magenta). Co-localization of vimentin and ILB4 markers is shown as white. Insets with dashed, white line are shown in the magnifications to the right. Insets with white line are shown in the magnifications to the bottom, showing zoomed-in areas with EndMT with nuclei stained with DAPI (blue). **f**, Quantification of vascular (podocalyxin^+^) Sca1^+^ area in the cerebellum of *Ccm3-iECKO* vehicle-treated (*n* = 13) and Cl-amidine-treated (10 mg kg^−1^ d^−1^; *n* = 8) mice. **g**, Quantification of vascular (podocalyxin^+^) vimentin^+^ area in the cerebellum of *Ccm3-iECKO* vehicle-treated (*n* = 12) and Cl-amidine-treated (10 mg kg^−1^ d^−1^; *n* = 8) mice. **h**, Representative images of the cerebellum of *Ccm3-iECKO* vehicle-treated mice (upper panel) and Cl-amidine-treated (10 mg kg^−1^ d^−1^) mice (lower panel) stained with Ki67 (magenta), ERG (green) and podocalyxin (white). Insets with dashed, white lines are shown in the magnifications to the right. **i**, Quantification of Ki67^+^ vascular (ERG^+^) area in the cerebellum of *Ccm3-iECKO* vehicle-treated (*n* = 13) and Cl-amidine-treated (10 mg kg^−1^ d^−1^; *n* = 8) mice. In the graphs, each data point represents one biological replicate; the bar indicates the median of each group; and the error bars represent the IQR. Statistical significance was determined using a Mann–Whitney *U*-test (two-tailed). cb, cerebellum. Source data

Activated vascular cells play a central role in inflammation and coagulation, and NETs were reported to promote endothelial cell activation^43^. We observed a significant decrease in the total ICAM-1 expression as well as the endothelial (ILB4^+^)-specific ICAM-1 expression after Cl-amidine treatment (*P* = 0.0465 and *P* = 0.0302, respectively) (Fig. 2a–c).

**Extended Data Fig. 3:**
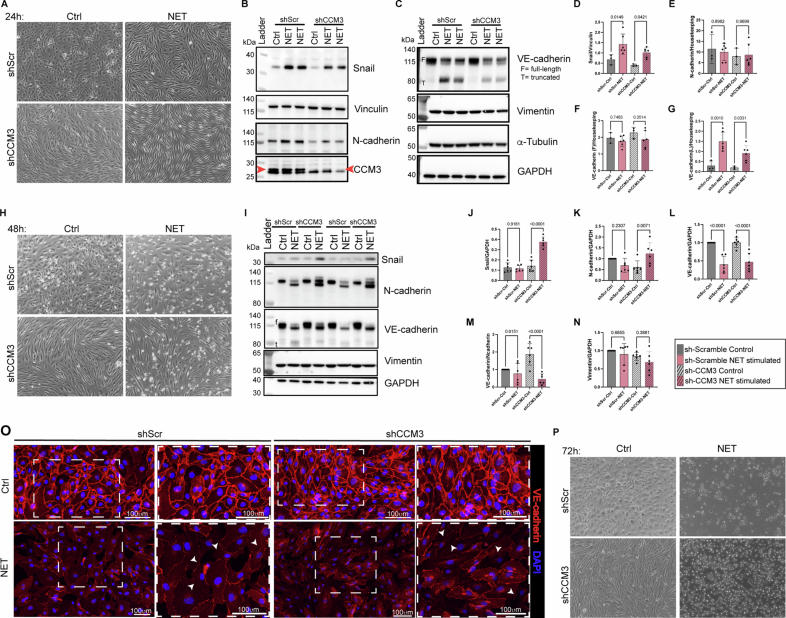
Effect of NET stimulation on HBMVECs. (**a**) Representative images of HBMVECs (shScramble (shScr), top and *shCCM3*, bottom) stimulated with 500 ng/mL NET-enriched supernatant or control (Ctrl) media for 24 h showing changes in cell morphology (n = 3). (**b–g**) Representative western blot images of HBMVECs (shscramble or shCCM3) stimulated with 500 ng/mL NET-enriched supernatant or control media for 24 h (n = 3 biological replicates) (**B**-**C**) and quantified (**D**-**G**; in E-G normalization was done to α-tubulin or GAPDH). (**h**) Representative images of HBMVECs (shScramble, top and *shCCM3*, bottom) stimulated with 500 ng/mL NET-enriched supernatant or control (Ctrl) media for 48 h showing showing changes in cell morphology (n = 6 biological replicates). (**i–n**) Representative western blot images of HBMVECs (shscramble or shCCM3) stimulated with 500 ng/mL NET-enriched supernatant or control media for 48 h (n = 6) (**J**) and quantified (**J**-**N**). (**o**) Representative images of HBMVECs (shScramble, left and *shCCM3*, right) stimulated with NET-enriched supernatant (bottom) or control (Ctrl; top) media for 48 h stained for VE-cadherin (red; junction) and DAPI (blue; nuclei). White arrow head highlights areas with no VE-cadherin staining. The stains were repeated independently three times with similar results. (**p**) Representative images of HBMVECs (shScramble, top and *shCCM3*, bottom) stimulated with 500 ng/mL NET-enriched supernatant or control (Ctrl) media for 72 h. The stains were repeated independently two times with similar results. In the graphs, the bar indicates the mean of each group, and the error bars represent the standard deviation. Statistical significance was determined using one-way ANOVA followed by a post-hoc Holm-sidak’s test. Source data

Continuous endothelial activation can induce EndMT^48^. EndMT is a known driver of CCM lesion progression^46^ and was reported to be promoted by NETs in vitro^45^. To ascertain if brain endothelial cells are pushed into EndMT by NETs, HBMVECs, wild-type or CCM3-deficient, were stimulated with NET-enriched supernatant. After 24 h of NET stimulation, we observed an elongated fibroblastoid morphology of the NET-stimulated cells (Extended Data Fig. 3a). In addition, the protein level of Snail—one of the master regulators of EndMT—was increased in both the shScramble and shCCM3 cells after NET stimulation (Extended Data Fig. 3b,d). Although no difference was observed in the mesenchymal markers vimentin and N-cadherin at this timepoint (Extended Data Fig. 3b,c,e and Supplementary Fig. 1), we observed an increase in the VE-cadherin intermediate fragment that is produced via proteolytic cleavage during endocytosis^49^ (Extended Data Fig. 3c,f,g and Supplementary Fig. 1). This suggested that a longer timepoint might be necessary to observe changes in mesenchymal markers. After 48 h of NET stimulation, increased protein level of Snail was observed only in the shCCM3 NET-stimulated cells (Extended Data Fig. 3h,i and Supplementary Fig. 2). Although there was still no difference in vimentin at this timepoint, there was a clear increase in N-cadherin and a decrease in VE-cadherin in the NET-treated cells (shScramble and shCCM3) (Extended Data Fig. 3i,j–n). The loss of VE-cadherin was also observed with immunofluorescence (Extended Data Fig. 3o). The cells died when stimulated with NETs for 72 h (Extended Data Fig. 3p), and, thus, a longer timepoint was not assessed.

These data suggest that NETs can promote EndMT in brain endothelial cells. We, therefore, questioned if NET inhibition via Cl-amidine could modulate this pathway. After Cl-amidine treatment, we assessed the expression of late EndMT markers (vimentin and Sca1)^46^. We observed a significant reduction in the endothelial-specific (podocalyxin^+^) Sca1 expression (*P* = 0.0302) (Fig. 2d,f) as well as a reduction in the endothelial-specific (ILB4^+^) vimentin expression upon Cl-amidine treatment (*P* = 0.0473) (Fig. 2e,g).

Endothelial cells that undergo EndMT become mesenchymal and, consequently, more proliferative and highly mobile^46^. This proliferative state is essential for the progression of CCM lesions. Thus, we assayed if Cl-amidine treatment affected proliferation. After Cl-amidine treatment, we observed a significant reduction in the area of proliferating endothelial cells measured by the vascular marker ERG, together with Ki67, within the cerebellum (*P* = 0.0018) (Fig. 2h,i). These data suggest that Cl-amidine rescued cells undergoing EndMT, and these endothelial cells, consequently, became less proliferative.

### Cl-amidine reduces anti-coagulant proteins in CCM

**Fig. 3:**
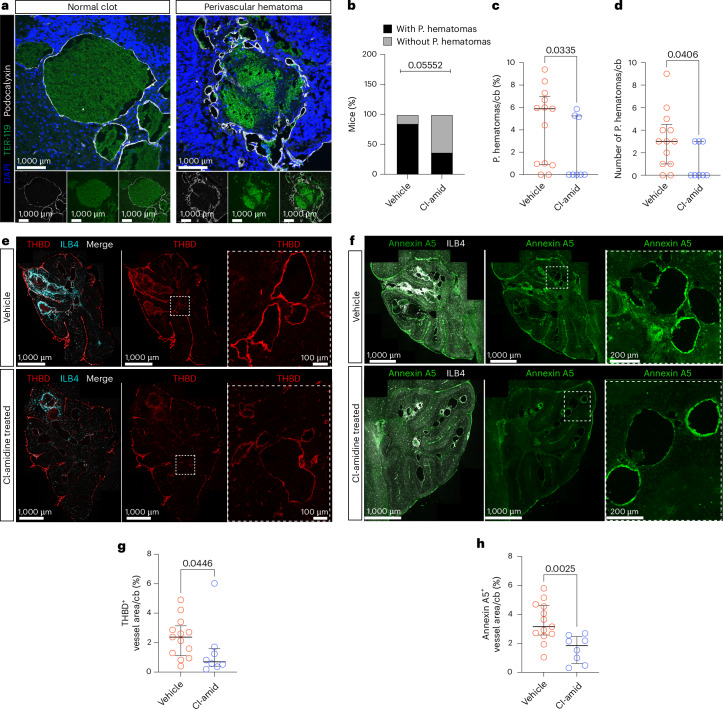
Cl-amidine reduced perivascular hematomas and anti-coagulant proteins in CCM. **a**, Representative images of a normal clot (left) and a perivascular hematoma (right) in the cerebellum of *Ccm3-iECKO* vehicle-treated mice stained with DAPI (blue), TER-119 (green) and podocalyxin (white). **b**, Quantification of percentage of mice with perivascular hematomas in *Ccm3-iECKO* vehicle-treated (*n* = 13) and Cl-amidine-treated (10 mg kg^−1^ d^−1^; *n* = 8) mice. Statistical significance was analyzed with Fisher’s exact *t*-test. **c**,**d**, Quantification of the number and area of perivascular hematomas in the cerebellum of *Ccm3-iECKO* vehicle-treated (*n* = 13) and Cl-amidine-treated (10 mg kg^−1^ d^−1^; *n* = 8) mice. **e**, Representative images of the cerebellum of *Ccm3-iECKO* vehicle-treated mice (upper panel) and Cl-amidine-treated (10 mg kg^−1^ d^−1^) mice (lower panel) stained with THBD (red) and ILB4 (cyan). Insets with dashed, white line are shown in the magnifications to the right. **f**, Representative images of the cerebellum of *Ccm3-iECKO* vehicle-treated mice (upper panel) and Cl-amidine-treated (10 mg kg^−1^ d^−1^) mice (lower panel) stained with annexin A5 (green) and ILB4 (white). Insets with dashed, white line are shown in the magnifications to the right. **g**,**h**, Quantification of vascular (ILB4^+^) THBD expression (**g**) and vascular (ILB4^+^) annexin A5 expression (**h**) in the cerebellum of *Ccm3-iECKO* vehicle-treated (*n* = 13) and Cl-amidine-treated (10 mg kg^−1^ d^−1^; *n* = 8) mice. In the graphs **c**,**d** and in **g**,**h**, each data point represents one biological replicate; the bar indicates the median of each group; and the error bars represent the IQR. Statistical significance was determined using a Mann–Whitney *U*-test (two-tailed). cb, cerebellum; P. hematoma, perivascular hematoma; THBD, thrombomodulin. Source data

**Extended Data Fig. 4:**
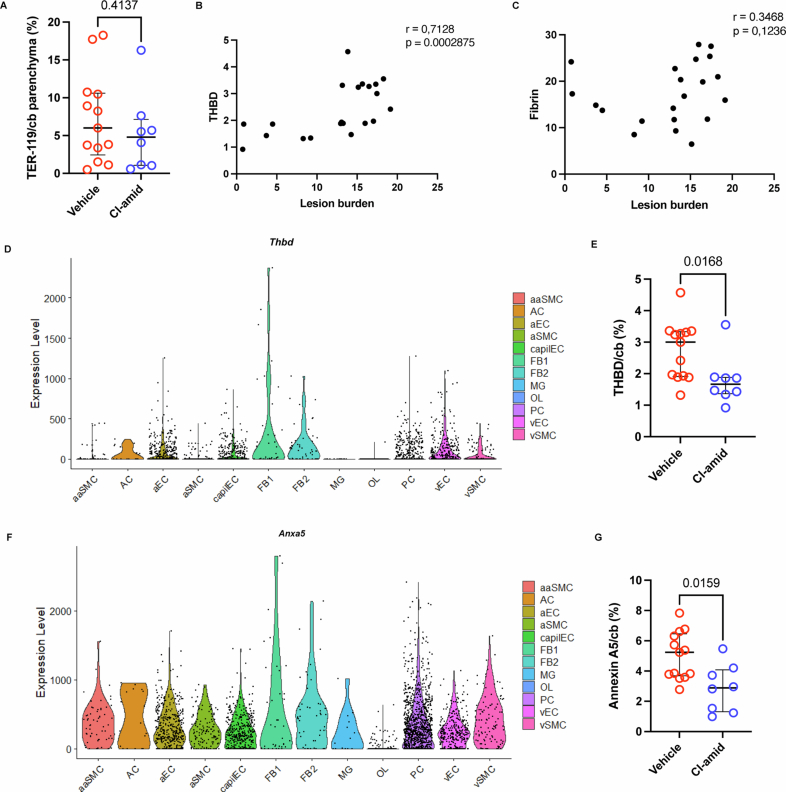
Thrombomodulin and Annexin A5 expression in mouse brain is not endothelial specific. (**a**) Quantification of TER-119^+^ leakage in the cerebellum parenchyma of *Ccm3*-iECKO vehicle (n = 13) and Cl-amidine (10 mg/kg/day; n = 8) treated mice. (**b, c**) Correlation analysis of thrombomodulin (THBD) **(b)** and fibrin **(c)** expression to lesion burden. **(d)** Gene expression of thrombomodulin (*Thbd*) in mouse brain vascular and perivascular cells **(e)** Quantification of total thrombomodulin (Thbd) expression in the cerebellum (cb) of *Ccm3-iECKO* vehicle and Cl-amidine (10 mg/kg/day) treated mice. **(f)** Gene expression of annexin A5 (*Anxa5*) in mouse brain vascular and perivascular cells. **(g)** Quantification of total annexin A5 expression in the cerebellum (cb) of *Ccm3-iECKO* vehicle (n = 13) and Cl-amidine (10 mg/kg/day; n = 8) treated mice. In the graphs (**A**, **E**, **G**), each data point represents one biological replicate, the bar indicates the median of each group, and the error bars represent the interquartile range. Statistical significance was determined using a Mann–Whitney U test (two tailed). Source data

Brain hemorrhage is one of the most severe symptoms experienced by patients with CCM^50^; thus, we examined the effect of Cl-amidine on bleeding in our *Ccm3-iECKO* mice. Using the erythrocyte marker TER-119, we investigated the erythrocyte leakage within the cerebellum. We observed no change in leaked TER-119 area (total bleeding area) within the cerebellum parenchyma upon Cl-amidine treatment (Extended Data Fig. 4a; *P* = 0.4137). We, however, observed bleeding hotspots that appeared to be perivascular hematomas within the brain parenchyma. These hematoma-like bleeds were characterized by a lake of erythrocytes surrounded by several small collateral vessels (Fig. 3a). There was a trend for fewer mice in the Cl-amidine-treated group having perivascular hematomas (*P* = 0.0555; Fig. 3b), and there was a reduction in the number and area of these perivascular hematomas after Cl-amidine treatment (*P* = 0.0406 and *P* = 0.0335, respectively) (Fig. 3c,d). To determine the possible rescue of the hematomas, a Fisherʼs exact *t*-test was used. The odds ratio for Cl-amidine-treated mice to have hematoma-like bleeds was 0.1091, indicating that there was a 89% reduced risk for hematoma-like bleeds after Cl-amidine treatment (Supplementary Table 1).

Increased thrombomodulin in CCM has been observed and reported to contribute to bleeding in CCM^10^. Interestingly, we observed a moderate correlation between lesion burden and thrombomodulin but not fibrin (*r* = 0.7128 and *r* = 0.3468, respectively) (Extended Data Fig. 4b,c). Another anti-coagulant protein, annexin A5, has also been reported to be highly expressed in CCM. The expression of the genes encoding these anti-coagulant proteins is not endothelial specific (Extended Data Fig. 4d,f)^39,40^; thus, we assessed the effect of Cl-amidine treatment in the total expression of these markers as well as in the endothelium. We observed a significant reduction in the total thrombomodulin area as well as the endothelial-specific thrombomodulin area within the cerebellum (*P* = 0.0168 and *P* = 0.0446, respectively) (Fig. 3e,g and Extended Data Fig. 4e). Additionally, Cl-amidine significantly reduced the total expression of annexin A5 in the cerebellum as well as specifically in the endothelium within the cerebellum (*P* = 0.0159 and *P* = 0.0025) (Fig. 3f,h and Extended Data Fig. 4g). This suggests that a mechanism through which Cl-amidine reduces perivascular hematoma is by modulating the anti-coagulant domain in CCM.

### Cl-amidine attenuates coagulation in CCM

**Fig. 4:**
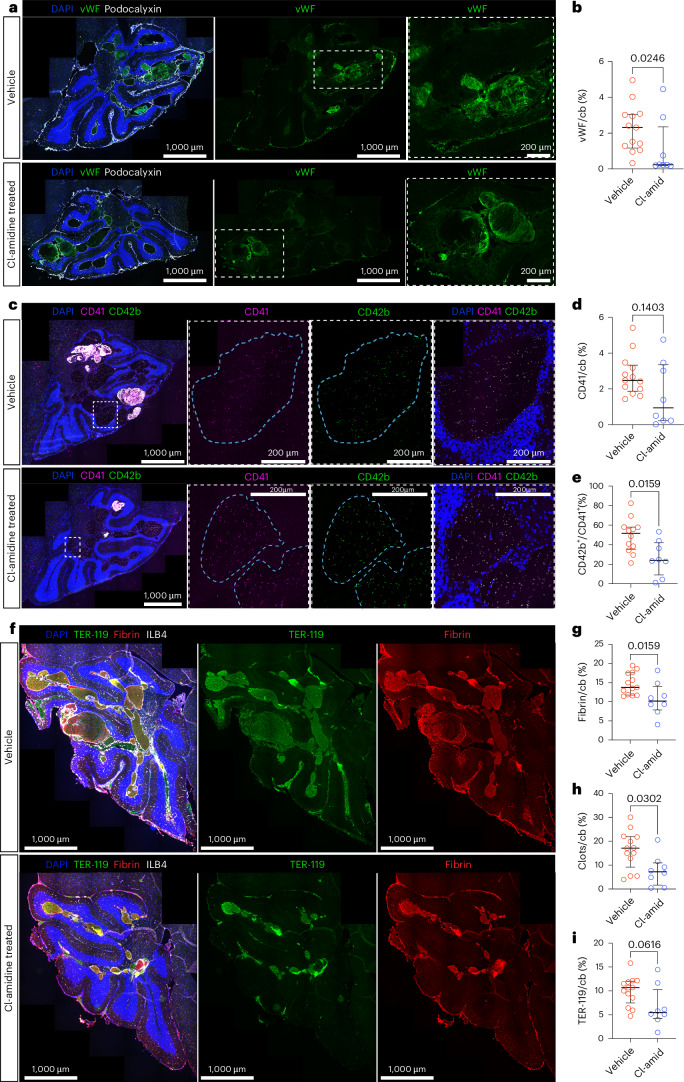
Cl-amidine reduced platelet activation and coagulation in CCM. **a**, Representative images of the cerebellum of *Ccm3-iECKO* vehicle-treated mice (upper panel) and Cl-amidine-treated (10 mg kg^−1^ d^−1^) mice (lower panel) stained with DAPI (blue), vWF (green) and podocalyxin (white). Insets with dashed, white line are shown in the magnifications to the right. **b**, Quantification of vWF^+^ area in the cerebellum of *Ccm3-iECKO* vehicle-treated (*n* = 13) and Cl-amidine-treated (10 mg kg^−1^ d^−1^; *n* = 8) mice. **c**, Representative images of the cerebellum of *Ccm3-iECKO* vehicle-treated mice (upper panel) and Cl-amidine-treated (10 mg kg^−1^ d^−1^) mice (lower panel) stained with DAPI (blue), CD41(magenta) and CD42b (green). Insets with dashed, white line are shown in the magnifications to the right (lesion periphery is shown with blue lines). **d**, Quantification of CD41^+^ area in the cerebellum of *Ccm3-iECKO* vehicle-treated (*n* = 13) and Cl-amidine-treated (10 mg kg^−1^ d^−1^; *n* = 8) mice. **e**, Quantification of total platelet (CD41^+^) area that is activated (CD42b^+^) in the cerebellum of *Ccm3-iECKO* vehicle-treated (*n* = 12) and Cl-amidine-treated (10 mg kg^−1^ d^−1^; *n* = 8) mice. **f**, Representative images of the cerebellum of *Ccm3-iECKO* vehicle-treated mice (upper panel) and Cl-amidine-treated (10 mg kg^−1^ d^−1^) mice (lower panel) stained with DAPI (blue), TER-119 (erythrocytes, green), fibrin (red) and ILB4 (white). **g**–**i**, Quantification of fibrin^+^ area (**h**), clot area (defined as lumens with fibrin and erythrocytes; **g**) and TER-119^+^ area (**i**), in the cerebellum of *Ccm3-iECKO* vehicle-treated (*n* = 13) and Cl-amidine-treated (10 mg kg^−1^ d^−1^; *n* = 8) mice. In the graphs, each data point represents one biological replicate; the bar indicates the median of each group; and the error bars represent the IQR. Statistical significance was determined using a Mann–Whitney *U*-test (two-tailed). cb, cerebellum. Source data

Dysregulated hemostasis in CCM was recently reported^8,10^. NETs were also reported to promote coagulation^51^; thus, we examined if Cl-amidine treatment affected the coagulation pathway. We observed a reduction in the expression of vWF after Cl-amidine treatment (*P* = 0.0246) (Fig. 4a,b). Because vWF mediates the activation of platelets^47^, we assessed the effect of Cl-amidine on platelets. We observed that, although the number of platelets remained the same (*P* = 0.1403), there was a significant decrease in the percent of activated platelets (CD42b^+^ cells) in the cerebellum of the Cl-amidine-treated mice compared to vehicle (*P* = 0.0159) (Fig. 4c–e). Furthermore, there was a significant reduction in the total fibrin^+^ area and areas of clots (defined as lumens with fibrin and erythrocytes) in the cerebellum in the Cl-amidine-treated mice compared to the controls (*P* = 0.0159 and *P* = 0.0302, respectively) (Fig. 4f–h). In addition, we observed a slight decrease in the total area filled with erythrocytes (TER-119^+^) (*P* = 0.0616) after Cl-amidine treatment (Fig. 4f,i).

### Cl-amidine rescues impaired clot breakdown in CCM

**Fig. 5:**
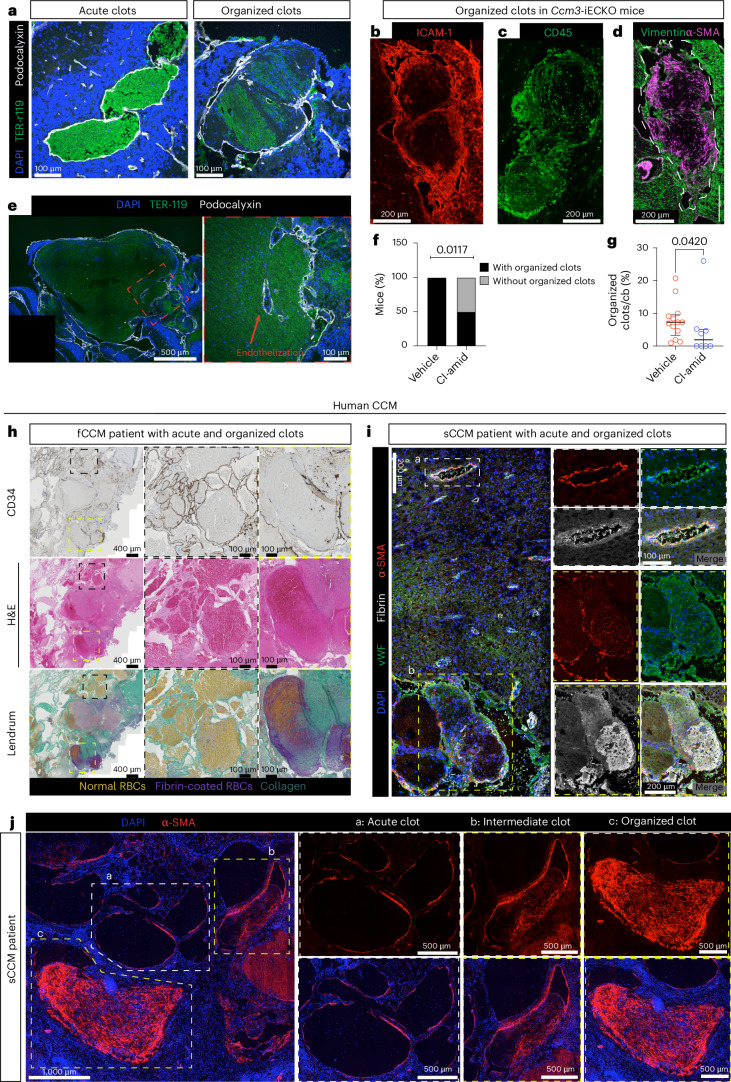
Impaired clot resolution in CCM is improved with Cl-amidine treatment. **a**, Representative images of acute (left) and chronic/organized (right) clots from the cerebellum of *Ccm3-iECKO* vehicle mice stained with DAPI (blue), TER-119 (green) and podocalyxin (white). **b**–**d**, Representative image of organized clots from the cerebellum of *Ccm3-iECKO* vehicle mice with increased ICAM-1 expression (red) (**b**), infiltrated with CD45^+^ cells (green) (**c**) and with increased expression of vimentin (green) and α-SMA (magenta) (**d**). **e**, Representative image showing endothelization of organized clots in the cerebellum of *Ccm3-iECKO* vehicle mice stained with DAPI (blue), TER-119 (green) and podocalyxin (white). Insets with red line highlight the magnifications to the right, with red arrow highlighting new vessels. **f**,**g**, Quantification of number of mice with organized clot (**f**) and organized clot area (**g**) in the cerebellum of *Ccm3-iECKO Ccm3-iECKO* vehicle-treated (*n* = 13) and Cl-amidine-treated (10 mg kg^−1^ d^−1^; *n* = 8) mice. Each data point represents one biological replicate; the bar indicates the median of each group; and the error bars represent the IQR. Statistical significance was determined using a Mann–Whitney *U*-test (two-tailed). **h**, Representative images of patients with familial CCM (fCCM) showing acute (black box) and organized (yellow box) clots. Samples were stained for CD34 (top panel), hematoxylin and eosin (middle panel) and Fraser–Lendrum stain (bottom panel). The stains were repeated independently in all samples (*n* = 3) with similar results. **i**, Representative images of acute (black box) and organized (yellow box) clots in sporadic CCM (sCCM) patient biopsy stained with DAPI (blue), vWF (green), α-SMA (red) and fibrin (white). Insets with white lines highlight the magnifications below. The stains were repeated independently in all samples (*n* = 4) with similar results. **j**, Representative images stained with DAPI (blue) and α-SMA (red) showing different clot stages from acute (white box; **a**) to intermediate (yellow box; **b**) and organized (yellow box; **c**) clots. Insets highlight the magnifications to the right. **a**: lesion without α-SMA deposition—acute clot; **b**: lesion with moderate α-SMA deposition—intermediate clot; **c**: lesion with intense α-SMA deposition—organized clot. The stains were repeated independently in all samples (*n* = 4) with similar results. cb, cerebellum. Source data

**Extended Data Fig. 5:**
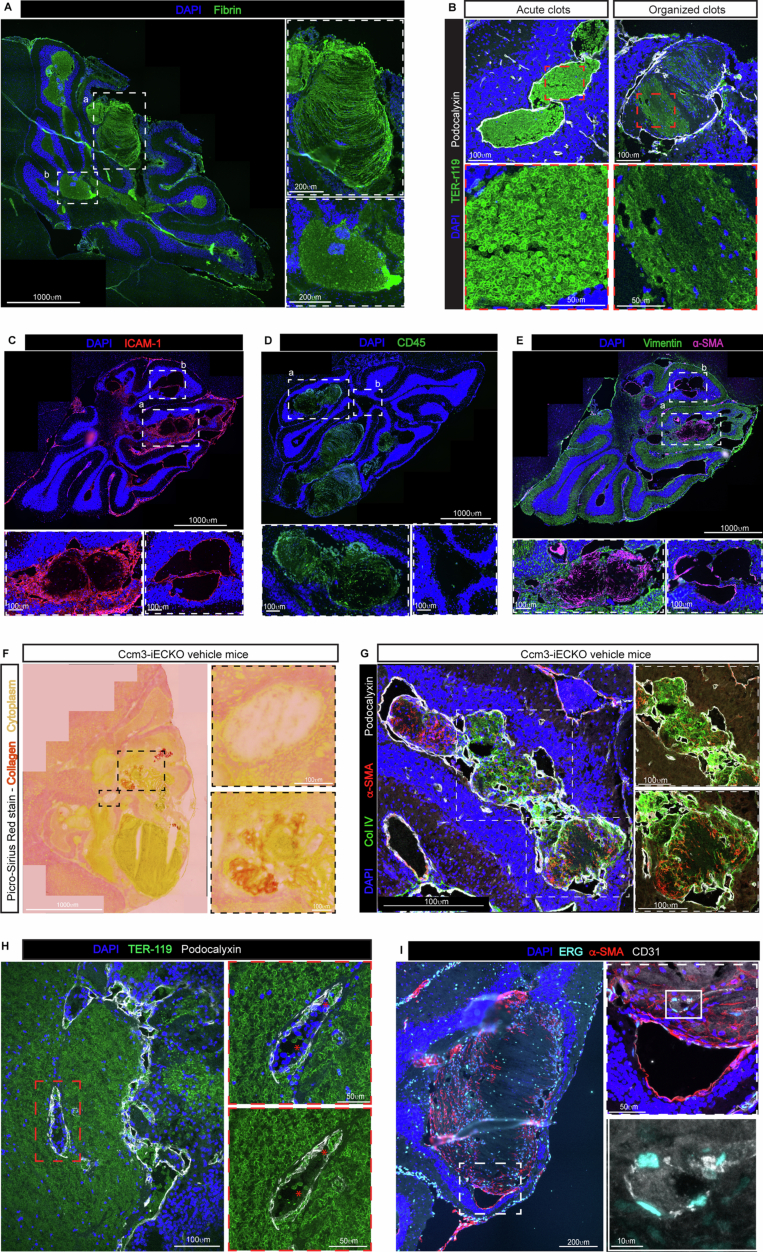
Characterization of organized clots in *Ccm3*-iECKO mice. (**a**) Representative images of the cerebellum of *Ccm3-iECKO* vehicle mice stained with DAPI (blue), and fibrin (green). Showing organized (**a**) and acute (**b**) clots. (**b**) Representative images of acute (left) and organized (right) clots the cerebellum of *Ccm3-iECKO* vehicle mice stained with DAPI (blue), TER-119 (green), and podocalyxin (white). Insets (bottom) are zoomed in regions. (**c**) Representative images of the cerebellum of *Ccm3-iECKO* mice stained with DAPI (blue), and ICAM-1 (red). Showing organized (**a**) and acute (**b**) clots. (**c**) Representative images of the cerebellum of *Ccm3-iECKO* vehicle mice stained with DAPI (blue), and CD45^+^ cells (in green). Showing organized (**a**) and acute (**b**) clots. (**d**) Representative images of the cerebellum of *Ccm3-iECKO* vehicle mice stained with DAPI (blue), vimentin (green) and α-SMA (magenta). Showing organized (**a**) and acute (**b**) clots. (**f**) Representative images of the cerebellum of *Ccm3-iECKO* vehicle mice stained with Picro-Sirius red stain (collagen-red/orange, cytoplasm-yellow) Insets with dashed, black line are shown in the magnifications to the right. (**g**) Representative images of the cerebellum of *Ccm3-iECKO* vehicle mice stained with DAPI (blue), collagen IV (Col IV; green), α-SMA (red) and podocalyxin (white). Showing increased Col IV deposition in organized clots. (**h**) Representative image showing endothelization of organized clots the cerebellum of *Ccm3-iECKO* vehicle mice stained with DAPI (blue), TER-119 (green), and podocalyxin (white). Insets (right) are zoomed in regions, with red asterisk highlighting red blood cells within the endothelized vessel. (**i**) Representative image showing endothelization of organized clots the cerebellum of *Ccm3-iECKO* mice at endpoint (P29) stained with DAPI (blue), α-SMA (red), CD31 (white) and ERG (cyan). Inserts (right) are zoomed in regions, highlighting an endothelized vessel. For all images (**a**-**i**), the stains were repeated independently in all the samples (n = 21) with similar results. Source data

After clot formation, fibrinolysis occurs to resolve the clots. Plasminogen activator inhibitor-1, an inhibitor of fibrinolysis, is upregulated in *Ccm3*-iECKO mice^8^ suggesting that fibrinolysis might be compromised in CCM. When regions containing thrombi were analyzed, we observed the presence of what appeared to be chronic clots. These were notable because, although fibrin-filled (Extended Data Fig. 5a), the erythrocytes within the clots were striated and threaded in appearance, with a partial reduction in the TER-119 stain intensity (Fig. 5a and Extended Data Fig. 5b).

We, therefore, sought to characterize the chronic/organized clots and found high expression of ICAM-1 in the lesions around these clots and high immune cell content (CD45^+^) (Fig. 5b,c and Extended Data Fig. 5c,d), indicating that these are regions of vascular activation. Our previously published bulk RNA-seq data showed that pathways related to wound healing are enriched when CCM3 is lost^8,24^. During wound healing, fibroblasts migrate to the clot to support clot resolution and, thereafter, undergo apoptosis^52^. Dysregulation of this process results in scarring or fibrosis. We observed the expression of vimentin and α-SMA, markers of fibroblasts and perivascular cells, respectively, present within the clots (Fig. 5d and Extended Data Fig. 5e), indicating that these clots are fibrotic with regions of wound healing. In fibrotic and organized clots, collagen deposition is increased^36,53^; indeed, we observed the presence of collagen in some of the organized clots (Extended Data Fig. 5f,g).

In some mice, we observed new vessels within the chronic clots, a process termed endothelization^54,55^ (Fig. 5e). Clot endothelization was observed in 100% of the mice with organized clots, and we observed erythrocytes within the vessels, indicating that they were functional (Extended Data Fig. 5h). The presence of functional vessels within these clots indicated that the clots had been present for a prolonged time and are likely regions of unresolved thrombi. Clot endothelization was also observed in older mice, suggesting that this is an ongoing attempt to re-perfuse the occluded vessel (Extended Data Fig. 5i).

**Extended Data Fig. 6:**
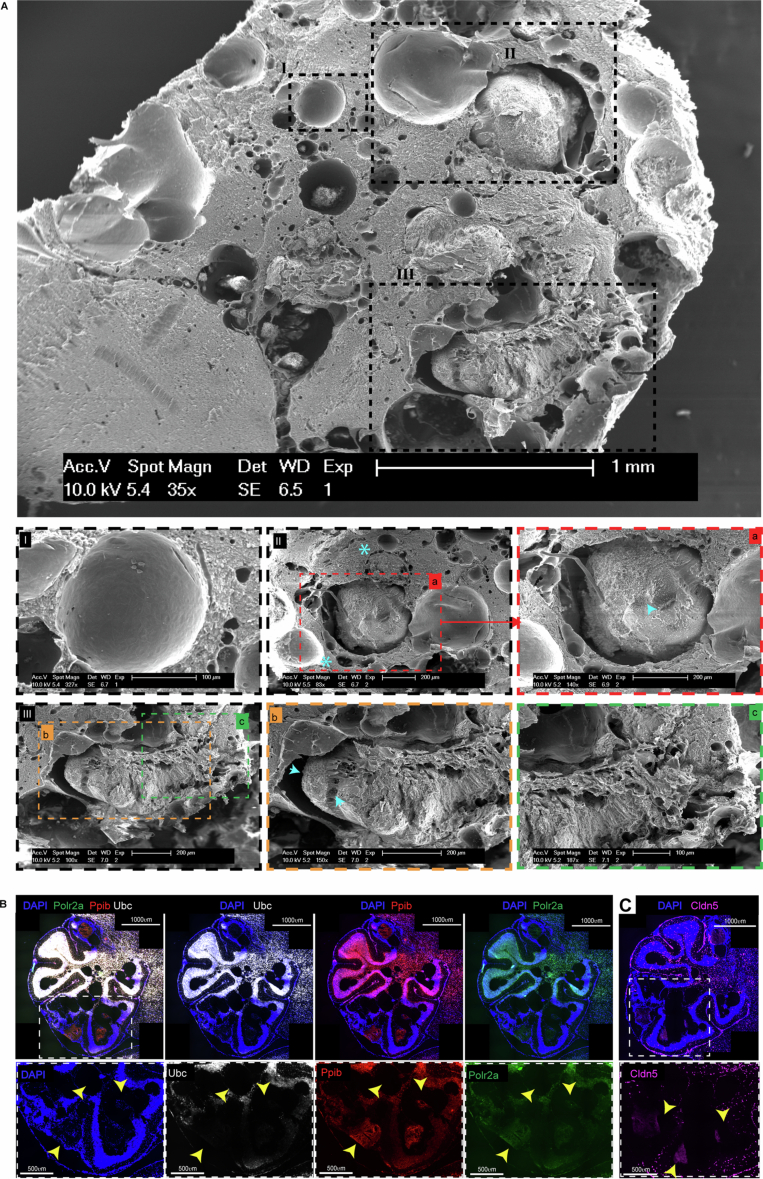
Organized clots in *Ccm3*-iECKO mice reduce vessel perfusion. (**a**): Representative scanning electron microscopy image of a perfused Ccm3-iECKO mice showing acute (I) and organized clot (II-III) areas. Insets at the bottom are close-up views of the dashed areas marked I-III. Inset figure (**a**) represents the red dashed area in figure II. Figure (**b**) from orange dashed area and figure (**c**) from green dashed area, both in figure III. Asterisks highlight collateral vessels. Arrow heads highlight erythrocytes. The stains were repeated independently in all the samples (n = 10) with similar results (**b**): Representative image of the cerebellum of *Ccm3-iECKO* vehicle mice stained via RNAscope with DAPI (blue), *Ubc* (white) *Polr2a* (green), *Ppib* (red) and *Cldn5* (magenta; consecutive section). Showing intense reduction of housekeeping genes around organized clots. The stains were repeated independently in all the samples (n = 10) with similar results. Source data

**Extended Data Fig. 7:**
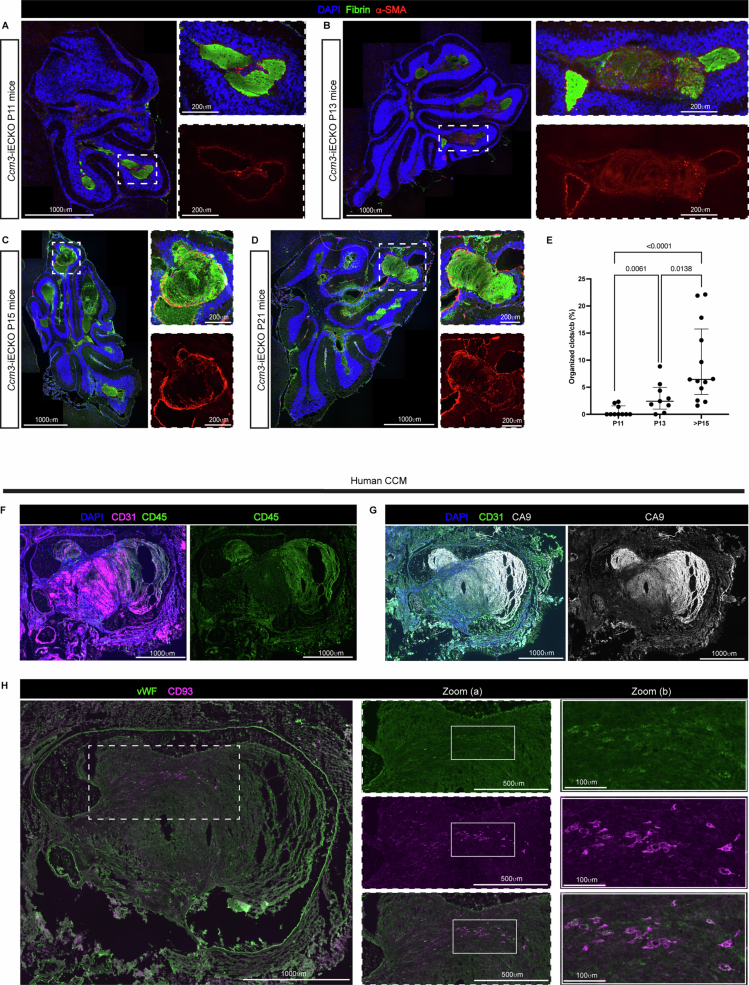
Kinetic analysis of clots in *Ccm3*-iECKO mice and characterization of organized clots in sporadic human CCM biopsies. (**a–d**) Representative images of *Ccm3*-iECKO mice aged P11 (No NETs; **a**), P13 (NETs present; **b**), P15 (**(c**), P21 (**d**), stained with DAPI (blue), fibrin (green) and α-SMA (red). (**e**) Quantification of organized clot area in the cerebellum (cb) of *Ccm3-iECKO* P11, P13, P15-28 mice. Each data point represents one biological replicate (n = 9-13 mice per group), the bar indicates the median of each group, and the error bars represent the interquartile range. Statistical significance was determined using a Kruskal-Wallis test followed by a post-hoc Mann-whitney test (**f**) Representative image of organized clots in sporadic human CCM stained with DAPI (blue), CD31 (magenta), and CD45 (green) showing CD45^+^ cell infiltration of organized clots. (**g**) Representative image of organized clots in sporadic human CCM stained with DAPI (blue), CD31 (green), and carbonic anhydrase (CA9, white) showing increased CA9 deposition in organized clots. (**h**) Representative image showing endothelization of organized clots in sporadic human CCM stained with vWF (green), and the angiogenesis marker, CD93 (magenta). Inserts with white line are shown in the magnifications to the right. For images **f**-**h**, the stains were repeated independently in all the samples (n = 4) with similar results. Source data

Further analysis of organized clots was performed using scanning electron microscopy on perfused *Ccm3*-iECKO mice. We observed the presence of organized clots in well-perfused mice, indicating that these organized clots likely reduce vessel perfusion (Extended Data Fig. 6a); additionally, we observed the presence of collateral vessels around organized clots. Based on these observations, we concluded that these clot structures are indeed organized chronic clots^36^. Using RNAscope, we observed a marked reduction in RNA puncta of housekeeping genes (*Polr2a*, *Ppib* and *Ubc)* around organized clots. Likewise, there was no *Cldn5* signal in the lesions containing organized clots (Extended Data Fig. 6b); these data indicate that the regions surrounding organized clots are negatively impacted and become unhealthy. The presence of NETs in clots can reduce fibrinolysis^27^. We previously showed that thrombi formation precedes NET formation, with NET formation beginning at postnatal day (P) 13 (ref. ^24^). Using the same samples from the study, we carried out a kinetic analysis of *Ccm3*-iECKO mice from P11 (thrombi present but NETs absent)^24^ to P13 (NETs present)^24^. Additional mice aged P15–P28 (disease progression and peak) were also analyzed. We observed that, although fibrin-filled clots were present at P11, there was an absence of organized clots in most of the mice (Extended Data Fig. 7a,e). The presence of organized clots was mostly observed starting at P13 *Ccm3*-iECKO mice (Extended Data Fig. 7b,e) with increasing perivascular cell (α-SMA^+^) recruitment into the clots as the mice aged (Extended Data Fig. 7c–e). We, therefore, examined the effect of Cl-amidine on these organized clots and found that the number of mice with organized clots in the treated group was significantly less than vehicle (*P* = 0.0117) (Fig. 5f). Furthermore, there was a significant reduction in the area of organized clots upon Cl-amidine treatment (*P* = 0.0420) (Fig. 5g).

**Extended Data Fig. 8:**
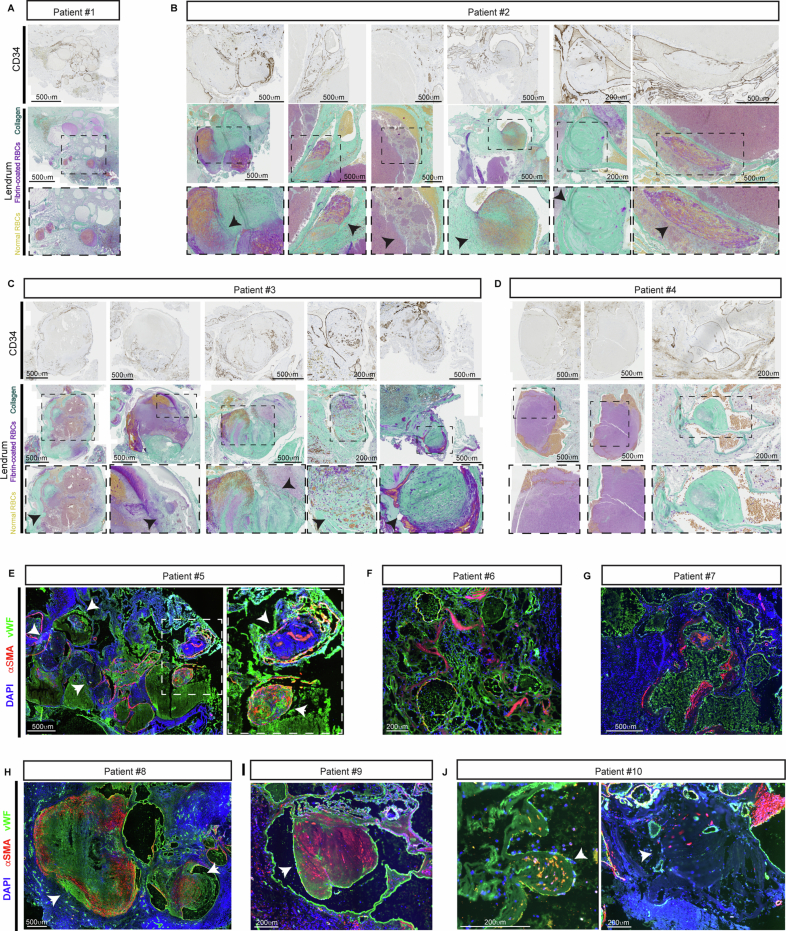
Organized clots in human CCMs. (**a–d**) Representative images of familial CCM (fCCM) patients showing acute or organized clots. Samples were stained for CD34 (top panel), and Fraser-Lendrum stain (normal red blood cells (yellow), fibrin coated red blood cells (purple), collagen (turquoise). Using Fraser-Lendrum stain, organized clots having increased collagen deposition are shown and contrasted with acute clots without collagen deposition. Insets with dashed, black line are shown in the magnifications below. Black arrows highlight the organized clots. (**e-j**) Representative images of sporadic CCM (sCCM) patients showing acute or organized clots in stained with DAPI (blue), vWF (green), α-SMA (red). Insets with dashed, white line are shown in the magnifications to the right. White arrows highlight the organized clots. For all images the stains were repeated independently in all the samples with similar results.

To determine the clinical significance of these organized clots, we analyzed their presence in human CCM tissue. Using the Fraser–Lendrum method, we investigated the presence of collagen-rich clots in familial CCM patient biopsy samples stained. In three of the four familial patients’ biopsies used in this study (Supplementary Table 2), we identified organized clots composed of increased collagen deposition (turquoise) compared to normal clots lacking collagen (Fig. 5h and Extended Data Fig. 8a–d). Further characterization of organized clots was carried out on snap-frozen sporadic CCM biopsies. We observed organized, fibrotic clots with a threaded appearance and increased α-SMA deposition within the clots (Fig. 5i) similar to that observed in the *Ccm3*-iECKO mice. Additionally, we observed clots at different stages in these patients—from acute clots to intermediate and fully organized clots (Fig. 5j). Within these clots, there were increased deposition of CD45^+^ cells (Extended Data Fig. 7f) in line with our murine findings, indicating that organized clots in CCM are sites of intense inflammation. Furthermore, we observed increased carbonic anhydrase (CA9), a marker of hypoxia^56^, within these clots (Extended Data Fig. 7g), suggesting that organized clots in human CCM are hypoxic. We also observed the presence of new vessels within the clots positive for the vascular angiogenic marker CD93 (refs. ^57,58^) (Extended Data Fig. 7h), suggesting endothelization also occurs in patients with CCM. We observed organized clots in four out of the six sporadic CCM samples analyzed (Supplementary Table 2 and Extended Data Fig. 8e–j).

### Cl-amidine reduces fibroblast activation in CCM

**Fig. 6:**
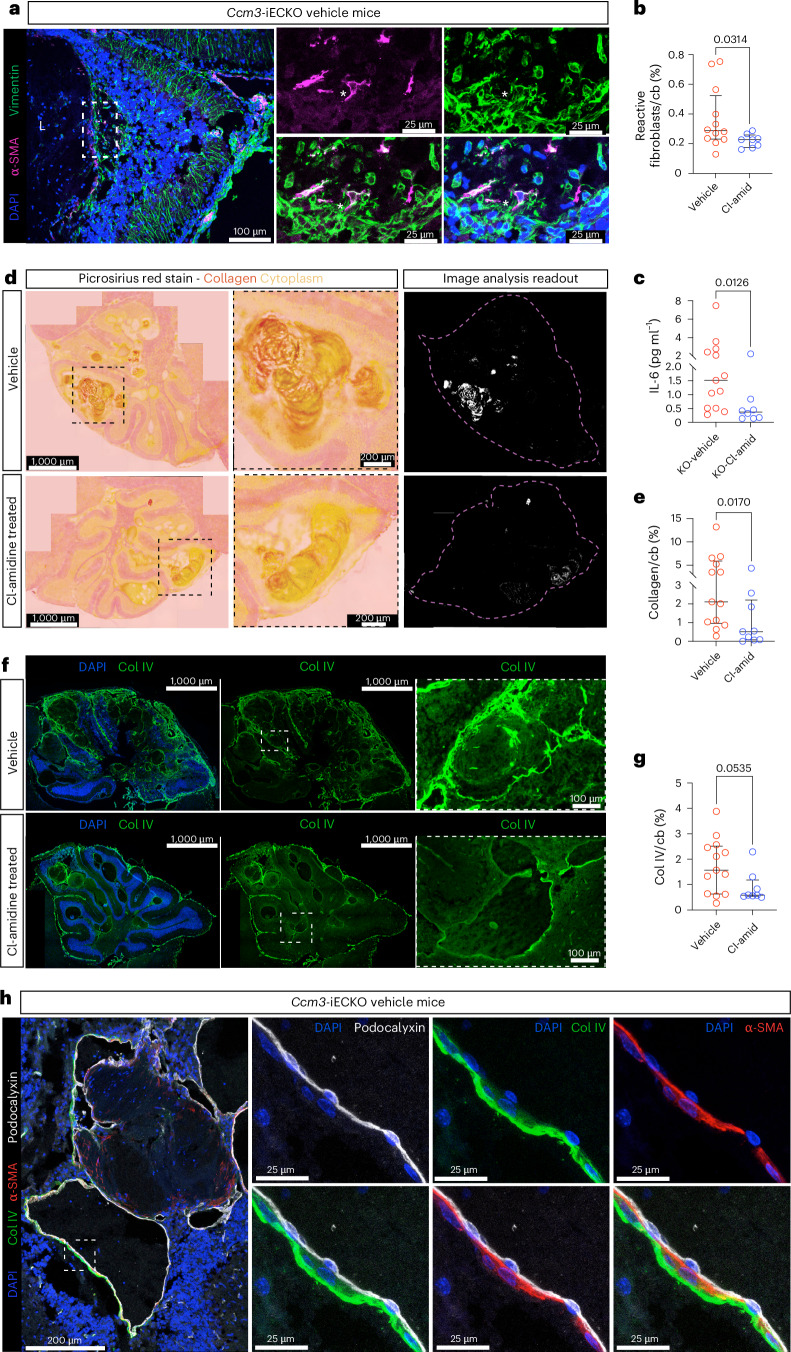
Cl-amidine treatment reduced Col IV deposition in the extracellular matrix and activated fibroblasts in CCM. **a**, Representative images of reactive fibroblasts in the cerebellum of *Ccm3-iECKO mice* stained with DAPI (blue), α-SMA (magenta) and vimentin (green). * represents reactive fibroblast expressing vimentin and α-SMA. **b**, Quantification of percentage of reactive fibroblast population in the cerebellum of *Ccm3-iECKO* vehicle-treated (*n* = 12) and Cl-amidine-treated (10 mg kg^−1^ d^−1^; *n* = 8) mice. **c**, Quantification of IL-6 levels in the plasma of *Ccm3-iECKO* vehicle-treated (*n* = 13) and Cl-amidine-treated (10 mg kg^−1^ d^−1^; *n* = 8) mice. **d**, Representative images of the cerebellum of *Ccm3-iECKO* vehicle-treated mice (upper panel) and Cl-amidine-treated (10 mg kg^−1^ d^−1^) mice (lower panel) stained with picrosirius red (collagen, red/orange; cytoplasm, yellow). Insets with dashed, black line are shown in the magnifications in the middle. Representation of the image analysis output is shown to the right. **e**, Quantification of collagen expression in the cerebellum of *Ccm3-iECKO* vehicle-treated (*n* = 13) and Cl-amidine-treated (10 mg kg^−1^ d^−1^; *n* = 8) mice. **f**, Representative images of the cerebellum of *Ccm3-iECKO* vehicle-treated mice (upper panel) and Cl-amidine-treated (10 mg kg^−1^ d^−1^) mice (lower panel) stained with DAPI (blue) and Col IV (green). Insets with dashed, white line are shown in the magnifications to the right. **g**, Quantification of total Col IV expression in the cerebellum of *Ccm3-iECKO* vehicle-treated (*n* = 13) and Cl-amidine-treated (10 mg kg^−1^ d^−1^; *n* = 8) mice. **h**, Representative images showing that Col IV is expressed in non-endothelial cells in *Ccm3-iECKO* mice stained with DAPI (blue), Col IV (green) and α-SMA (red). The stains were repeated independently in all samples (*n* = 21) with similar results. In the graphs, each data point represents one biological replicate; the bar indicates the median of each group; and the error bars represent the IQR. Statistical significance was determined using a Mann–Whitney *U*-test (two-tailed). cb, cerebellum. Source data

As described above, we observed the recruitment of fibroblasts into organized clots and the presence of collagen IV (Col IV) within some of them. Fibroblasts, in response to multiple stimuli^59^, can become activated—a state characterized by the upregulation of α-SMA^60^. An association between NETs and fibroblast activation was reported in lung tissue^60^. Thus, we examined the fibroblast population in *Ccm3*-iECKO mice and observed the presence of activated fibroblasts. We characterized activated fibroblasts as vimentin^+^/α-SMA^+^/ILB4^−^ cells (Fig. 6a, asterisk) to specifically exclude endothelial cells (ILB4^+^) undergoing EndMT. We observed a significant reduction in the population of activated fibroblasts upon Cl-amidine treatment (*P* = 0.0314) (Fig. 6b).

During inflammation, the release of IL-6 can promote fibroblast activation and, consequently, increase fibrosis^61^. We assessed if this cytokine was affected by Cl-amidine treatment. In line with our previously published data on IL-6 expression in neuroinflammation^24^, we observed here an increase in IL-6 plasma levels in the *Ccm3*-iECKO vehicle mice (median concentration, 1.515 pg ml^−1^) compared to the *Ccm3-*WT vehicle controls (median concentration, 0.1390 pg ml^−1^) (*P* = 0.0017). This increase was attenuated upon Cl-amidine treatment (*P* = 0.0126) (Fig. 6c).

A thickened BM is a hallmark of CCM lesions^62^, which may reduce the physical interaction between endothelial cells and other cells within the neurovascular unit^63^. Fibroblasts are key producers of BM matrix, including collagens in different organs^64,65^, and activated fibroblasts were linked to increased collagen deposition in renal fibrosis^66^. Activated fibroblasts are known to contribute to brain fibrosis^67^, raising the possibility that they might be responsible for the thickened BM observed in CCM. Using picrosirius red stain, we analyzed the collagen deposition within the cerebellum and observed a significant reduction in collagen in the Cl-amidine-treated group compared to vehicles (Fig. 6c,d). Furthermore, using Col IV as a marker of the BM, we observed a trend for decreased Col IV expression after Cl-amidine treatment (*P* = 0.0535) (Fig. 6e,f). Although endothelial cells can produce Col IV (ref. ^68^), we observed that more of the Col IV signal was deposited closer to α-SMA^+^ cells and not the podocalyxin^+^ endothelium layer (Fig. 6g). This suggests that perivascular cells, such as fibroblasts, are responsible for the increased Col IV in CCM.

### Cl-amidine reduced neuroinflammation in CCM

**Fig. 7:**
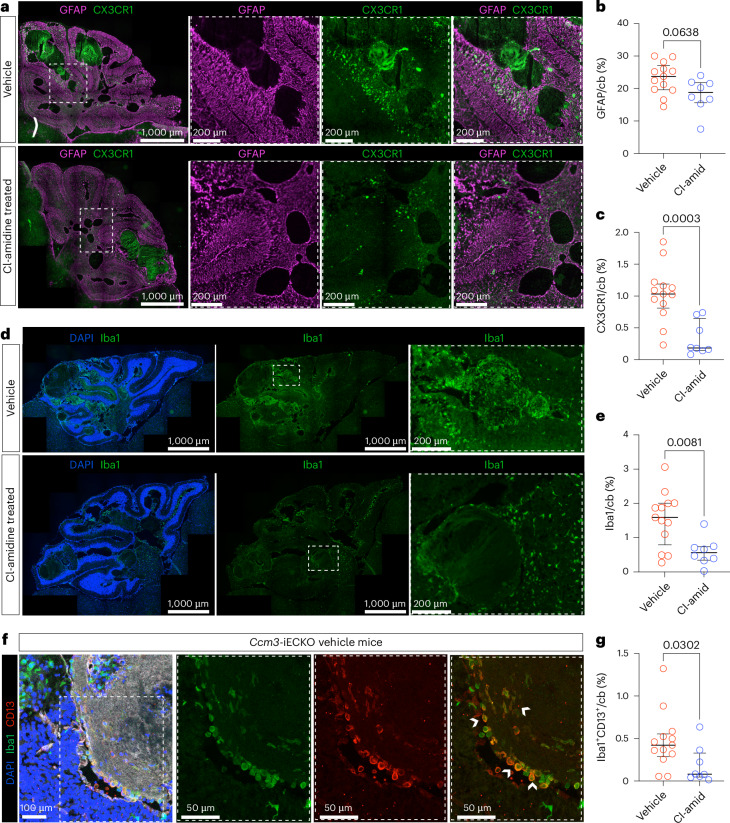
Cl-amidine reduced neuroinflammation in CCM. **a**, Representative images of the cerebellum of *Ccm3-iECKO* vehicle-treated mice (upper panel) and Cl-amidine-treated (10 mg kg^−1^ d^−1^) mice (lower panel) stained with GFAP (magenta) and CX3CR1 (green). Insets with dashed, white line are shown in the magnifications to the right. **b**, Quantification of GFAP expression in the cerebellum of *Ccm3-iECKO* vehicle-treated (*n* = 13) and Cl-amidine-treated (10 mg kg^−1^ d^−1^; *n* = 8) mice. **c**, Quantification of CX3CR1 expression in the cerebellum of *Ccm3-iECKO* vehicle-treated (*n* = 13) and Cl-amidine-treated (10 mg kg^−1^ d^−1^; *n* = 8) mice. **d**, Representative images of the cerebellum of *Ccm3-iECKO* vehicle-treated mice (upper panel) and Cl-amidine-treated (10 mg kg^−1^ d^−1^) mice (lower panel) stained with DAPI (blue) and Iba1 (green). Insets with dashed, white line are shown in the magnifications to the right. **e**, Quantification of Iba1 expression in the cerebellum of *Ccm3-iECKO* vehicle-treated (*n* = 13) and Cl-amidine-treated (10 mg kg^−1^ d^−1^; *n* = 8) mice. **f**, Representative images of reactive microglia in the cerebellum of *Ccm3-iECKO* mice stained with DAPI (blue), CD13 (red) and Iba1 (green). Insets with dashed, white line are shown in the magnifications to the right. White arrows represent reactive microglia expressing Iba1 and CD13. **g**, Quantification of percentage of reactive microglia in the cerebellum of *Ccm3-iECKO* vehicle-treated (*n* = 13) and Cl-amidine-treated (10 mg kg^−1^ d^−1^; *n* = 8) mice. Insets (far right) are zoomed-in regions. In the graphs, each data point represents one biological replicate; the bar indicates the median of each group; and the error bars represent the IQR. Statistical significance was determined using a Mann–Whitney *U*-test (two-tailed). cb, cerebellum. Source data

The recruitment of microglial cells by reactive astrocytes via C-X3-C motif chemokine receptor 1 (CX3CR1) into active CCM lesions was recently reported to promote the growth of lesions, bleeding and immunothrombosis^14^. We, therefore, investigated if the reduction in lesion burden observed with Cl-amidine was related to activation of astrocytes or microglia. The astrocyte marker GFAP is increased upon astrocyte activation^44^; hence, we assessed if GFAP and, consequently, reactive astrocytes were affected by Cl-amidine treatment. We observed a trend of decreased GFAP after Cl-amidine treatment (*P* = 0.0638; Fig. 7a). There was, however, a significant reduction in CX3CR1 with Cl-amidine treatment (*P* = 0.0003; Fig. 7c).

Furthermore, we observed a significant reduction in microglial cell populations, using the microglia pan-marker Iba1, upon Cl-amidine treatment (*P* = 0.0081) (Fig. 7d,e). During brain injury, microglial cells can become activated and start expressing CD13 (ref. ^69^). We observed that activated microglial cells (Iba1/CD13 double-positive cells) were present in *Ccm3*-iECKO mice, and this population was significantly reduced by Cl-amidine (*P* = 0.0302) (Fig. 7f,g). This suggests that a mechanism through which Cl-amidine ameliorates lesion burden is by reducing astrocyte/microglia-mediated neuroinflammation.

## Discussion

In this study, we investigated the role of NETs in the pathogenesis and progression of CCM by inhibiting NET formation using Cl-amidine. We previously reported the presence of NETs in both human and murine CCM and showed that degrading extruded NETs using DNase I in *Ccm3*-iECKO mice reduced endothelial leakage^24^. Our previous study showed that neutrophil peak activity occurs between P11 and P13 (ref. ^24^); thus, in the present study, mice were treated with Cl-amidine from P8 to P15. Cl-amidine has high specificity for PAD4 (the main neutrophil PAD) and preferentially inhibits it over PAD2 (refs. ^31,32^). Cl-amidine would inhibit PAD activity regardless of the cell expressing it, including vascular cells. A study had shown that citrullination in mesenteric venules resulted in increased release of vWF strings^37^. In our study, HBMVECs treated with Cl-amidine showed no changes in vWF string release, indicating that citrullination did not occur in brain vascular cells. Additionally, we showed that PADs are absent in murine brain vascular and perivascular cells and that this does not change when CCM3 is lost. These results indicate that the effects observed upon Cl-amidine treatment in mice are majorly through its ability to inhibit neutrophil PAD4 and, consequently, NET formation. However, we cannot exclude the possibility that some effect may be due to Cl-amidine's pleiotropic inhibition of other PADs.

In the present study, we found that inhibiting NET formation with Cl-amidine, blocks citrullination and NET formation, reduced lesion burden and influenced key pathways known to be dysregulated in CCM, such as endothelial activation, EndMT, immunothrombosis and neuroinflammation. Cl-amidine has a capacity to affect several physiological activities, such as inhibition of inflammation, through the modulation of immune cells and reducing immunothrombosis^70^. Although other weaker PAD inhibitors, such as paclitaxel or streptomycin, have been used in the treatment of various diseases^71^, Cl-amidine has not yet not reached clinical application. With its potential in inhibiting immunothrombosis and normalization of the brain microenvironment, it may serve as a potential treatment in CCM. In this study, *Ccm3*-iECKO mice in both treatment and control groups suffered adverse effects (death or balance issues). There were, however, no adverse effects seen in *Ccm3*-WT mice treated with Cl-amidine, suggesting that the adverse effects observed were more likely due to the disease burden or spleen issues that occur in the full-body endothelial cell CCM3 knockout mice used in this study^72^. However, inhibiting NETs could affect immunity, and this must be carefully addressed in a clinical setting.

CCM is a disease where endothelial cell homeostasis is disrupted, resulting in increased cellular activation. Our study showed that when NET formation is inhibited, endothelial activation is decreased. Continuous endothelial activation induces EndMT^48^, which, in turn, can drive lesion progression in CCM^46^. Based on other studies on the effect of NETs on endothelial cells^43^, a concentration of 500 ng ml^−1^ NET was used to stimulate brain endothelial cells (wild-type and CCM3-deficient, because CCM lesions comprise both mutant and wild-type cells^12^). After 24-h NET stimulation in vitro, we observed that shScramble and shCCM3 brain endothelial cells became elongated with a fibroblastoid morphology. Furthermore, the expression of Snail, a master regulator of EndMT, was increased, indicating that NETs induce EndMT in both wild-type and CCM3-mutant brain endothelial cells. A 48-h NET stimulation showed an increase in the mesenchymal marker N-cadherin and a decrease in VE-cadherin. This suggests that NETs have the capacity to induce EndMT in brain endothelial cells, at least to some extent. This is in line with other in vitro studies showing that NETs promote EndMT^45^. NETs might also have an indirect effect on EndMT via its effect on platelet activation^51^, as activated platelets produce TGF-β, which is a potent inducer of EndMT^46^^,73^. After Cl-amidine treatment in mice, we observed a reduction in the EndMT markers Sca1 and vimentin. The transition to a mesenchymal phenotype causes a highly proliferative state in endothelial cells^46^; this, in turn, can promote lesion progression. We observed reduced endothelial proliferation when NET formation was inhibited. These data suggest that the attenuation of the endothelial activation–EndMT-proliferation axis could be one mechanism through which Cl-amidine treatment reduced lesion burden in CCM.

Dysregulation of both pro-coagulation and anti-coagulation pathways in CCM was reported^8,10^, and the upregulation of the anti-coagulant pathway was linked to increased risk for bleeding in CCM^10^. Additionally, NETs were shown to promote clot formation^51^. In our study, Cl-amidine treatment reduced the expression of anti-coagulant proteins thrombomodulin and annexin A5. Although total bleeding (measured as erythrocytes within the cerebellum parenchyma) was unchanged, we observed a reduction in bleeding spots, termed ‘perivascular hematomas’, in the brain. Cl-amidine treatment also attenuated the pro-coagulation pathway, resulting in reduced platelet activation and clot formation. This suggests that NETs promoted the dysregulation of the pro-coagulant and anti-coagulant pathways in CCM. Thus, when inhibited with Cl-amidine, the hemostatic system can rebalance. We previously showed that coagulation precedes NET formation in our mouse model^24^; it was, therefore, not unexpected that inhibiting NET formation did not completely stop coagulation. This suggests that a combination therapy targeting both NETs and coagulation might be even more beneficial for CCM.

Furthermore, in the present study, we showed and characterized the presence of chronic/organized clots, which are regions of unresolved thrombi and wound healing. Using multiple methods, we observed the presence of organized clots in both murine and human (familial and sporadic) CCMs. As described^36^, we observed increased immune cell recruitment to these clots, indicating that they are sites for inflammation in the brain. During clot organization, fibroblasts are recruited into the clots, contributing to fibrosis and collagen deposition^36^. Indeed, we observed increased fibroblast recruitment and collagen deposition in both human and murine CCMs. In sporadic CCMs, we observed clots at different stages of organization: acute clots without perivascular cell recruitment, to intermediate clots with some perivascular cells, to fully organized clots filled with perivascular cells^36^. In our mouse model, we observed increased perivascular cell recruitment as the mice aged. This suggests that clot organization in CCM is an ongoing process, with these clots becoming increasingly fibrotic over time.

Clot fibrosis can limit vessel perfusion. Indeed, using scanning electron microscopy, we observed that these organized clots remained even after rigorous perfusion. The presence of the hypoxic marker CA9 in organized clots of human CCMs also suggests reduced vessel perfusion, as indicated by this hypoxia marker. We also observed that RNA levels of housekeeping genes and Claudin 5 were reduced around the organized clots, indicating that the organized clots destabilize and negatively impact the surrounding tissue. Based on these findings, we conclude that coagulation is unresolved in CCM, leading to the formation of organized clots. As a result, these organized clots limit perfusion and contribute to hypoxia, likely causing neural cell damage and sustained inflammation. In addition, they will hinder access of therapeutics to obstructed areas within the brain. Thus, therapies that focus on removing these organized clots would presumably be beneficial for CCM. The presence of NETs in clots is known to reduce fibrinolysis. Interestingly, in murine CCM, we observed organized clots only after NET formation had begun. When NET formation was inhibited with Cl-amidine, the number and size of organized clots were reduced, implicating NETs in the formation and growth of organized clots in CCM. Although it might sound counterintuitive to propose anti-thrombotic therapies for a disease with a risk for hemorrhage, studies have suggested that anti-thrombotic medications can help reduce the risk of intracranial hemorrhage or focal neurologic deficits that are associated with CCM^74^. It is important to note that the Cl-amidine-treated mice that had reduced organized clots did not have increased bleeding.

Activated fibroblasts have been implicated in fibrosis in various contexts, such as wound healing and abnormal matrix remodeling, chronic inflammation and tumor metastasis^75,76^. We observed the presence of fibroblasts in organized clots, suggesting that they promote clot fibrosis in CCM. We observed that a subset of fibroblasts in CCM becomes activated and expresses α-SMA. Inhibiting NET formation reduced fibroblast activation, suggesting that NETs promote fibroblast activation in CCM. This is line with a recent study where Cl-amidine reduced lipopolysaccharide -induced inflammation in gingival fibroblasts^77^. This reduction of activated fibroblasts may be one of the mechanisms through which Cl-amidine promotes resolution of organized clots in CCM, because the clots then become less fibrotic and more prone to fibrinolysis.

Furthermore, when activated, fibroblasts become highly secretory and upregulate the production of cytokines, extracellular matrix components and remodeling factors^78^, such as collagens. Using picrosirius red stain, we observed a reduction in collagen deposition after NET inhibition. A thickened BM in CCM lesions is a known phenomenon^62^; this thickening reduces the ability of endothelial cells to physically interact with pericytes and other cells in the neurovascular unit, consequently affecting their function. We observed that inhibiting NET formation reduced the deposition of Col IV within the BM. Although Col IV can be expressed by endothelial cells, we observed that more of the Col IV signal was deposited closer to α-SMA^+^ cells and not the podocalyxin^+^ endothelium layer. This suggests that perivascular cells, such as fibroblasts, are responsible for the increased Col IV in CCM. Indeed, Col IV is produced in high amounts by fibroblasts^64,65^, and RNA-seq of human CCM showed that fibroblast-like cells were the strongest contributors to increased collagen deposition in CCM^79^. NETs were reported to increase the production of collagen and other extracellular matrix components in activated lung fibroblasts^60^. This suggests that, in CCM, NETs are involved in the activation of fibroblasts and increases their secretion of collagens, such as Col IV, contributing to a thickening of the BM.

The recruitment of microglial cells into active CCM lesions, driven by reactive astrocytes via CX3CR1, was reported to promote lesion progression, bleeding and immunothrombosis^14^. In the present study, when NET formation was inhibited, reactive astrocytes, CX3CR1 and microglia cells were reduced. In addition, we showed the presence of a subset of activated microglia (CD13^+^Iba1^+^ cells) in CCM. This subtype of microglia was reported to be present only during neural injury^69^. In our study, microglial activation was reduced when the formation of NETs was inhibited. Granule proteins released during NET formation are able to recruit and activate other inflammatory cells^30^. Based on this, we hypothesize that, after NET formation, microglia cells are recruited either directly or via reactive astrocytes into CCM lesions. These microglia cells then become activated by granule proteins released during NET formation and promote lesion progression in CCM. Thus, our data suggest that NETs play a crucial role in inducing neuroinflammation in CCM.

**Fig. 8:**
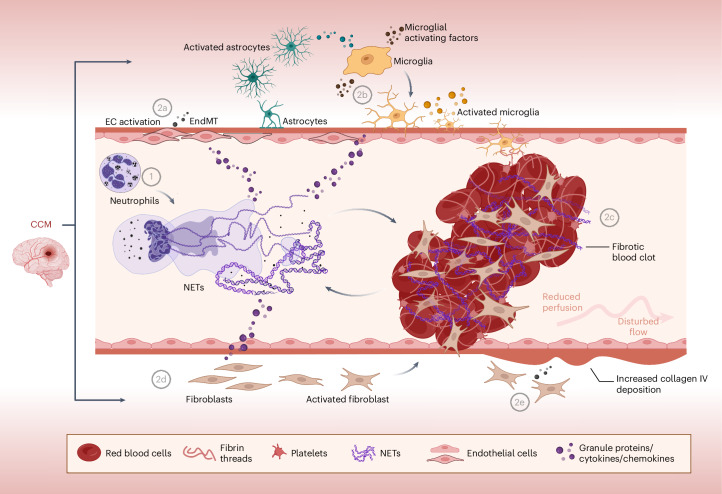
Graphical summary of findings. Representative summary of the findings of this study. In CCM, neutrophils become activated, leading to the release of NETs (1). These NETs trigger or exacerbate processes dysregulated in CCM: endothelial activation and EndMT (2a), neuroinflammation (2b), coagulation and clot organization (2c), fibroblast activation (2d) and increased collagen deposition (2e). The graphical abstract was created with BioRender.

In summary, in this study, we see that CCM represents a convergence of dysregulated processes involving multiple cell types, exerting an impact on the brain. Our findings reveal that the presence of NETs exacerbates these processes, potentially through the direct harmful effect of granule proteins and cytokines released during NET formation or by recruiting and activating reactive cell types (Fig. 8). This leads to the creation of an environment that fosters lesion growth and neurovascular dysfunction (Fig. 8). Notably, NETs were shown to promote a pro-malignant environment and metastasis in cancer^27^. Consequently, inhibition of NET formation rebalances the CCM microenvironment, promoting endothelial homeostasis and creating a healthier environment that hinders lesion progression.

## Methods

### Ethics approval

All experiments involving animals were conducted according to the principles of the Swedish National Board for Laboratory Animals and the European Convention for Animal Care. Animal experiments were approved by the regional ethics committees in Uppsala, Sweden (5.8.18-16224-2020).

### Genetically modified mice and disease induction

Endothelial-specific *Ccm3*-deficient C57BL/6J mice (*Cdh5*(PAC)-Cre-ER^T2^/*Ccm3*^flox/flox^) were generated by crossing *Cdh5*(PAC)-Cre-ER^T2^ mice with *Ccm3*^fl/fl^ mice (Taconic Artemis). The details of this were previously described. Tamoxifen (Sigma-Aldrich, T5648) was dissolved in ethanol and further diluted in corn oil to a concentration of 2 mg ml^−1^. To induce the disease as previously described, the pups were injected intragastrically with 5 μg of tamoxifen at P1. In this study, the Ccm3-deficient mice are termed *Ccm3*-iECKO mice. Cdh5(PAC)-Cre-ER^T2^/*Ccm3*^flox/flox^ Cre-negative pups, or *Cdh5*(PAC)-Cre-ER^T2^/*Ccm3*^flox/flox^ Cre-positive pups injected with corn oil, were used as wild-type controls (hereafter termed *Ccm3*-WT). All mice were housed in microisolator cages containing wood shavings and enrichment. They were kept in a climate-controlled environment with a 12-h light/dark cycle. Mice were fed standard rodent chow with free access to water and regularly monitored for health status.

### Cl-amidine treatment strategy

Cl-amidine (N-[(1S)-1-(aminocarbonyl)-4-[(2-chloro-1-iminoethyl)amino]butyl]-benzamide, monohydrochloride, CAS no. 1373232-26-8; Cayman Chemicals, cat no.10599, batch no. 0583527-26) was dissolved in DMSO to a concentration of 25 mg ml^−1^. This was then further diluted in 1× PBS to a final concentration of 5 mg ml^−1^. Treatment groups were assigned randomly among littermates. The inhibition of NETs with 10 mg kg^−1^ Cl-amidine was previously established^32,34^. Thus, in the treatment group, 10 mg kg^−1^ Cl-amidine was administered from P8 to P14/15 once daily via intraperitoneal injection. Control (vehicle) mice received DMSO diluted fivefold with 1× PBS. Mice were euthanized 6 h after the final Cl-amidine injection. Details on all mice used in the study are available in Supplementary Table 3.

### Animal information

**Extended Data Fig. 9:**
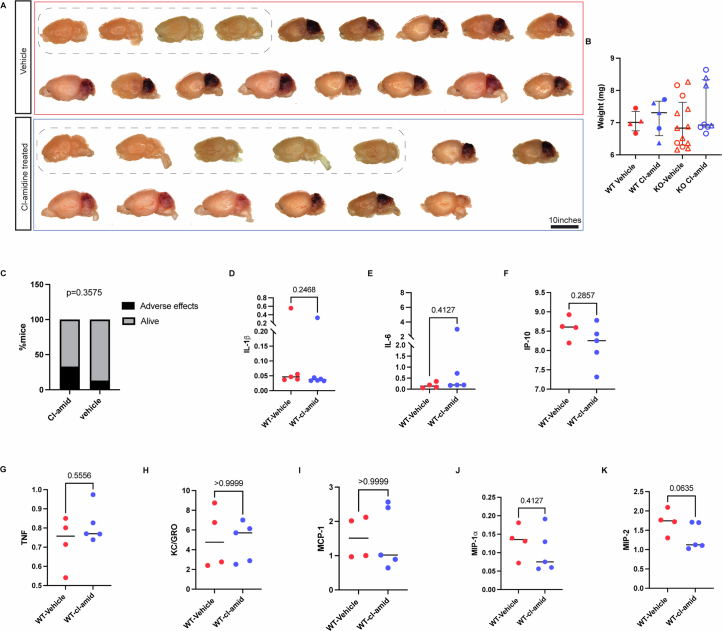
Effect of NET inhibition on mouse weight, and cytokines. (**a**) Macroscopic images of mouse brains used in study. Brains within the box with dashed lines are wild type mice. (**b**) Quantification of mouse weight and sex distribution across Cl-amidine treated vs vehicle groups. Open and filled circles represent females and open and filled triangles represent males. (**c**) Quantification of adverse effects (death or balance issues) in *Ccm3*-iECKO mice used in the study. (**d–k**) Quantification of IL-1β (**d**), IL-6 (**e**), IP-10 (**f**), TNF (**g**), KC/GRO (**h**), MCP-1 (**i**), MIP-1α (**j**), MIP-2 (**k**) levels in the plasma of *Ccm3-*WT vehicle and Cl-amidine (10 mg/kg/day) treated mice.

In total, 36 mice were included in the study, both males and females (Extended Data Fig. 9a); a detailed data table on all mice used is presented as Supplementary Table 3. Two *Ccm3*-iECKO mice from the vehicle group and three *Ccm3*-iECKO mice from the treatment group died before the end of the experiment. One mouse from the *Ccm3*-iECKO treatment group was excluded from the analysis because it had unusual balance issues on the day of collection. There was, however, no change in mice weights between the treatment groups (*P* = 0.5179; Extended Data Fig. 9b), indicating that the Cl-amidine treatment did not affect the mice adversely. To study if the dose given was toxic, *Ccm3*-WT mice were treated with Cl-amidine, and no adverse effects on their health, weight and total well-being were observed (Extended Data Fig. 9b). Furthermore, a Fisher’s test comparing the number of animals with adverse effects or death with those that survived in both treatment groups showed no significant difference (*P* = 0.3575; Extended Data Fig. 9c). In line with this, cytokine analysis of inflammatory cytokines (IL-1β, IL-6, IP-10, KC/GRO, MCP-1, MIP-1α, MIP-2 and TNF) showed no difference in the wild-type mice treated with Cl-amidine or vehicle (Extended Data Fig. 9d–k). This suggests that the *Ccm3*-iECKO mice died due to effects of the disease rather than the treatment.

### Quantitative cytokine assay from plasma

Blood samples were obtained from mice via cardiac puncture, using acid-citrate-dextrose buffer (38 mM L^−1^ citric acid, 75 mM L^−1^ trisodium citrate, 100 mM L^−1^ dextrose) as the anti-coagulant. Plasma was isolated by centrifugation at 4,500*g* for 15 minutes at 4 °C. The plasma was aliquoted and frozen at −80 °C until analysis. The plasma concentration of IL-6, IL-1β, IP-10, KC/GRO, MCP-1, MIP-1α, MIP-2 and TNF was determined using a U-Plex Mouse Cytokine kit (Meso Scale Discovery, K15069M-1) according to the manufacturer’s instructions. Total plasma protein concentrations were determined using BCA Protein Assay kits (Thermo Fisher Scientific, 23225).

### Tissue preparation

Mouse brains were extracted from the skulls and cut sagitally. One half was embedded in optimal cutting temperature (OCT) compound (Thermo Fisher Scientific) and snap frozen using dry-ice-cold isopentane. The other half was immersed in 4% paraformaldehyde (PFA) and fixed overnight at 4 °C. The fixed brains were washed with 1× PBS, immersed in 30% sucrose and kept at 4 °C until they sunk to the bottom of the tube. Stereomicroscope (Leica, M205 FA) images of the brains were obtained, after which the brains were embedded in OCT and frozen with dry ice. All samples were stored at −80 °C until sectioning. Cryosections (7 μm) were cut using a cryostat (Thermo Fisher Scientifc, CryoStar NX50), and the sections were stored at −20 °C until analysis.

### Immunofluorescence and microscopy

PFA-fixed cryosections were rinsed in 1× PBS for 10 min to rehydrate the samples, whereas snap-frozen cryosections were fixed in ice-cold methanol for 10 min and left to air dry. Sections (PFA-fixed or snap-frozen) were rehydrated for 10 minutes using PBS + 0.05% Tween 20 (PBST). After rehydration, samples were blocked, permeabilized and stained with primary antibody (listed in Supplementary Table 4) using a solution of 0.5% Triton-X and 10% donkey serum in 1× PBS. The samples were incubated overnight at 4 °C. The next day, they were washed 3 × 5 minutes with PBST and incubated with secondary antibody for 1 h at room temperature. The samples were once again washed, counterstained with 10 µg ml^−1^ DAPI (Molecular Probes) and mounted using Fluoromount-G (Thermo Fisher Scientific).

For GLUT1, CX3CR1, Ly6G and GFAP, the staining was carried out as described above with the following changes: unfixed frozen sections were used for the staining; primary antibody incubation was done at room temperature for 2 h; and the slides were fixed with 4% PFA for 5 minutes and rinsed with PBS before being mounted.

Microscopy of all sections was done using a Leica DMi8 microscope and a Leica SP8 confocal microscope.

### RNAscope

Snap-frozen tissue was cryosectioned (10 μm) and used for RNAscope following the manufacturer’s instructions (ACD Bio, RNAscope Multiplex Fluorescent Reagent Kit v2 Assay) with the following changes: fixation with PFA was done for 25 minutes, and pre-treatment was done with protease III for 20 minuntes. Probes from ACD Bio were used to detect the genes encoding for *Polr2a*, *Ppib*, *Ubc* and *Cldn5* (Claudin 5). Microscopy of all sections was done using a Leica DMi8 microscope.

### Picrosirius red stain

Snap-frozen cryosections were fixed in ice-cold acetone for 10 minutes and left to air dry. The sections were then hydrated in water for 5 min and incubated in the picrosirius red stain solution (Abcam, ab150681) for 30 min. The slides were dipped twice in acetic acid solution, dehydrated 3 × 1 minute with 100% ethanol and cleared in xylene for 5 minutes. Samples were mounted using Pertex. Microscopy of all sections was done using a Leica DMi8 microscope. Collagen-positive signals appear red.

### Scanning electron microscopy

*Ccm3*-iECKO mice were deeply anesthetized by an intraperitoneal injection of Avertin (tribromoethanol) and perfused with electron microcopy fixative containing 2% PFA, 2.5% glutaraldehyde (Electron Microscopy Sciences), 2% sucrose and 2 mmol L^−1^ CaCl_2_ in PBS pH 7.4. After perfusion, mice were kept in a sealed plastic bag at room temperature for 2 h. The cerebellum was excised and post-fixed in EM fixative for 72 h at 4 °C. Samples were then transferred to PBS and sectioned at 1–2-mm intervals using a mouse brain matrix (Zivic Instruments, BSMAS005-1 and BSMAS005-2), and a stereomicroscope (Wild Heerbrugg) was used to observe the dissection. The sections were post-fixed in 1% unbuffered osmium tetroxide for 1 h. Afterwards, the sections were washed in the buffer three times, dehydrated through a series of graded ethanol and critical point dried in an Agar E3000 critical point dryer (Quorum Technologies) using liquid CO_2_ as the drying agent. The sections were then mounted and coated with gold using an Emitech K550X sputter device. Observations were made using a Philips XL 30 ESEM operated at 10-kV accelerating voltage with images recorded digitally.

### Human CCM brain biopsies

Biopsies from patients with sporadic CCM were collected at the Department of Neurosurgery, Helsinki University Hospital (Finland) during their routine surgical treatment, where the decision for surgery was based solely on the patient’s clinical needs. Paraffin-embedded biopsies from patients with familial CCM were donated by the patients directly to the Alliance to Cure Cavernous Malformation CCM Biobank after advertisements by the patient organization, Alliance to Cure Cavernous Malformation. The biopsies were obtained for this study through a material transfer agreement. The patients gave their consent for use of biopsies for research studies. The patient cohort included 10 patients with CCMs (age 3–66 years; male:female ratio 6:4; sporadic:familial ratio 6:4; Supplementary Table 2). Collection and use of the samples in research was approved by organizational and ethical committees: Helsinki University Hospital (HUS/125/2018), the Committee on Research Ethics of Helsinki University Hospital (HUS/3648/2017) and the Swedish Ethical Review Authority (EPM; 2019-04715, 2019-06374 and 2017-165).

### Staining of human CCM biopsies

For immunofluorescence, frozen cryosections were fixed in 4% PFA for 15 minutes and rinsed with PBS. Sections were rehydrated for 10 minutes using PBST. After rehydration, samples were blocked and stained with primary antibody (listed in Supplementary Table 4) using a solution of 0.5% Triton-X and 10% donkey serum in 1× PBS. The samples were incubated overnight at 4 °C. The next day, they were washed 3 × 10 minutes with PBST and incubated with secondary antibody for 1 h at room temperature. The samples were once again washed, counterstained with 10 µg ml^−1^ DAPI (Molecular Probes) and mounted using Fluoromount-G (Thermo Fisher Scientific).

For Fraser–Lendrum staining, formalin-fixed paraffin-embedded (FFPE) biopsies from patients with familial CCM were stained according to standard routine protocols.

### Bioinformatics analysis for gene expressions

The violin plots of *Padi4* and *Padi2* (genes encoding PAD4 and PAD2) expression in mouse brain were generated with the Seurat package for R from publicly available datasets on mouse cerebellar single-nucleus RNA-seq^38^ and mouse brain vascular and perivascular single-cell RNA-seq^39,40^. The violin plots for *Thbd* and *Anxa5* (genes encoding thrombomodulin and annexin A5) expression in mouse brain were generated with the seurat package from R from publicly available datasets on mouse brain vascular and perivascular single-cell RNA-seq^39,40^.

### shCCM3 Lentivirus infection

Primary HBMVECs were purchased from iXCells Biotechnologies (10HU-051) at passage 1 (p1). The cells were seeded on dishes coated with Collagen I and maintained in Endothelial Cell Growth Medium MV 2 (Lonza) without antibiotics. Lentivirus vectors were prepared by transfecting Lenti-X 293T cells (Takara, 632180) with third-generation lentiviral system plasmids. After 48 h, the supernatant was collected and centrifuged for 2 h at 20,000*g*. The pellet containing the viral particles was resuspended in ice-cold sterile 1% BSA/PBS and stored at −80 °C. Semi-confluent HBMVECs were transduced with lentivirus containing short hairpin RNA (shRNA) against human CCM3 (Santa Cruz Biotechnology, sc-62084) for 24 h. The viruses were removed by adding fresh medium, and the cells were cultured for an extra 48 h in complete medium. Transduced cells were selected with 2 μg ml^−1^ puromycin. After selection, cells were trypsinized and expanded for experiments.

### Cl-amidine treatment of HBMVECs

HBMVECs, shScramble and shCCM3, were seeded (70,000 cells) on dishes coated with Collagen I and maintained in Endothelial Cell Growth Medium MV 2 (PromoCell). The half-maximal inhibitory concentration (IC_50_) value for the inhibition of PAD4 by Cl-amidine is 5.9 μm (ref. ^31^). Thus, cells were treated with either 6-μm Cl-amidine (Cayman Chemicals, cat. no. 10599, batch no. 0583527-26) or DMSO and cultured for 24 h. The cells were then fixed with 4% PFA (room temperature) for 20 minutes and washed with 1× PBS.

### NET isolation

Anti-cogulated (EDTA) blood (14mL) was obtained from healthy volunteers. Neutrophils were isolated using a MACSxpress Whole Blood Neutrophil Isolation kit (Miltenyi Biotec, 130-104-434) according to the manufacturer’s instructions. The isolated neutrophils were centrifuged at 300*g* for 10 minutes, and the pellet was resuspended with 2 ml of RPMI medium/3% FBS. Neutrophils (1 ml per well) were seeded into six-well cell culture plates and stimulated with 500 nM phorbol myristate acetate for 4 h at 37 °C. The supernatant was discarded, and wells were gently washed with 3 ml of Dulbecco-modified PBS (D-PBS) and treated with 10 U µl^−1^ of the restriction enzyme DNase I (Roche, 11284932001) and 2 mM CaCl_2_ in RPMI media for 20 minutes at 37 °C. Then, 5 mM EDTA was added to stop DNase activity. The supernatant was collected and centrifuged at 300*g* for 5 minutes at 4 °C to remove whole cell and debris. The cell-free, NET-rich supernatant was collected and stored at −80 °C. The DNA concentration was measured using PicoGreen (Thermo Fisher Scientific) according to the manufacturer’s instructions.

### NET stimulation of HBMVECs

HBMVECs p6–8, shScramble and shCCM3, were seeded (300,000 cells) on six-well dishes (Sarstedt, 83.3920) pre-coated with Collagen I and maintained in Endothelial Cell Growth Medium MV2 (PromoCell) until confluency. The cells were then stimulated with 500 ng ml^−1^ cell-free, NET-rich supernatant at 37 °C for 24–72 h, after which they were collected for further analysis.

### Western blot

HBMVECs were rinsed twice with cold DPBS and lysed with ice-cold RIPA buffer (Thermo Fisher Scientific, 89901), supplemented with protease and phosphatase inhibitors (Thermo Fisher Scientific, 87785). The protein concentration of the cell lysates was determined with the Pierce BCA Protein Assay kit (Thermo Fisher Scientific, 23225). Samples were prepared for western blot with a homemade 6× SDS sample buffer and boiled for 5 minutes at 95 °C. The samples were then loaded onto a 1.5-mm 4–12% BisTris pre-cast gel (NuPAGE) and run with a MOPS running buffer (Thermo Fisher Scientific) for 15 minutes at 80 V and then for 1 h at 130 V. The Page Ruler Prestained Protein Ladder (Thermo Fisher Scientific, 26616) was used as molecular weight marker. Proteins were dry transferred onto a PVDF membrane with the iBLOT 2 Gel Transfer Device (Thermo Fisher Scientific). The membrane was blocked for 1 h at room temperature with 5% BSA/TBS-T, incubated with the primary antibodies (listed in Supplementary Table 4) diluted in 5% BSA/TBS-T overnight on a rocker at 4 °C, washed with TBS-T and then incubated with the secondary antibodies diluted in 5% BSA/TBS-T for 1 h at room temperature. The membrane was washed with TBS-T and then TBS and then incubated with ECL Prime Western Blotting Detection Reagents (GE Healthcare, RPN2232) and then exposed under a transilluminator (Thermo Fisher Scientific, iBright CL1500). iBright software (version 5.3.0) was used to analyze the images.

### Immunofluorescence for HBMVECs

PFA-fixed cells were permeabilized with 0.1% Triton X-100 in PBS for 5 minutes and then rinsed twice with PBS. The cells were incubated for 30 minutes with blocking buffer (3% BSA diluted in PBS). The primary antibodies (listed in Supplementary Table 4) were diluted in fresh blocking buffer and incubated overnight at 4 °C in a humid chamber. Thereafter, the cells were washed three times with PBS and incubated in secondary antibodies (Thermo Fisher Scientific) diluted in blocking buffer for 1 h at room temperature. The cells were washed three times with PBS, counterstained with 10 µg ml^−1^ DAPI (Molecular Probes) for 5 minutes and mounted with Fluoromount-G.

### Image analysis

Analysis of microscopy images was carried out using Fiji (ImageJ). To assess protein expression, a cutoff threshold was set to select positive signals, after which the percent area of the positive signal within the cerebellum was quantified. Threshold values were determined manually and independently for each experiment. To semi-automatically assess lesion burden, we quantified the visibly bloody regions of stereo microscopy images, with the idea that area of lesions visible on the surface of the brains would correlate to disease severity. The macro used color thresholding of Hue, Saturation and Brightness to automatically detect these visibly bloody regions from images of mouse brain hemispheres, as viewed sagittally. Hue, Saturation and Brightness parameters were determined manually and independently for each experiment. To identify rarer cell types (reactive fibroblasts and activated microglia) or vessel-specific expression, a macro was designed to automatically identify and quantify areas with co-localization of the relevant proteins.

### Statistical analysis

Data on Cl-amidine treatment are presented as median values ± interquartile range (IQR). Statistical analysis was performed with GraphPad Prism 8.0.2 (GraphPad Software). To test for significance between two groups, a Mann–Whitney *U*-test (two-tailed) was performed. For testing in groups of more than two, a Kruskal–Wallis test was performed, followed by a post hoc Mann–Whitney *U*-test (two-tailed). Correlation analyses were done using a Spearman’s correlation test. Fisher’s exact *t*-test was used to test proportions. To test the relationships between effects, logistic regression and nonlinear regression analyses were used.

For in vitro studies, data are presented as mean values ± s.d. For testing in groups of more than two, a one-way ANOVA test was performed, followed by a post hoc Sidak’s test. *P* values less than 0.05 were considered statistically significant.

### Inclusion and ethics statement

Data are reported in conformity with Animal Research: Reporting of In Vivo Experiments (ARRIVE) guidelines.

### Reporting summary

Further information on research design is available in the Nature Portfolio Reporting Summary linked to this article.

## Supplementary information

Supplementary information Supplementary Figs. 1 and 2 containing full blots for Fig. 3c (panel 4) and Fig. 3i (panel 1).

Reporting Summary

Supplementary Tables 1–4

## Source data

Source Data Figs. 1–7. A single file containing all statistical source data, with clearly named tabs for each figure.

Source Data Extended Data Figs. 1–7. A single file containing all statistical source data, with clearly named tabs for each extended data figure.

Source Data Extended Data Fig. 3. A zip folder containing unprocessed western blots for Extended Data Fig. 3.

## Data Availability

Bulk endothelial cell sequencing (GEO: GSE246373), single-cell RNA sequencing (GEO: GSE155788; GSE98816, GSE99058 and GSE99235) and single-nucleus RNA sequencing (GSE165371) data were retrieved from previously published studies. Source data are provided with this paper. Figures that have associated raw data include Figs. 1b,c,e,f, 2b,c,f,g,i, 3b–d,g,h, 4b,d,e,g–i, 5f,g, 6b,c,e,g and 7b,c,e,g and Extended Data Figs. 1a,b,e, 2b,c,l,m, 3d–g,j–n, 4a–c,e,g, 7e and 9b–k.

## Code availability

All custom codes used for image analysis of this study are available upon reasonable request from the corresponding author (P.U.M.) and can be found on GitHub: 10.5281/zenodo.10590829.
