# Supplementary material for: Pharmacological blocking of neutrophil extracellular traps attenuates immunothrombosis and neuroinflammation in cerebral cavernous malformation
Source: Nat Cardiovasc Res. 2024 Dec 4;3(12):1549–67. doi: 10.1038/s44161-024-00577-y (PMC11634782; doi:10.1038/s44161-024-00577-y)

nature cardiovascular research

Supplementary information https://doi.org/10.1038/s44161-024-00577-y

Pharmacological blocking of neutrophil extracellular traps attenuates immunothrombosis and neuroinflammation in cerebral cavernous malformation

In the format provided by the authors and unedited

Table of Contents

Supplementary Fig 1: Uncropped blots for Figures 3c (panel 1 and 4)....................2

Supplementary Fig 2: Uncropped blots for Figures 3i (panel 1) 2

Source data file for Figure 1A:..3

Source data file for Figure 1B:..3

Source data file for Figure 2: 3

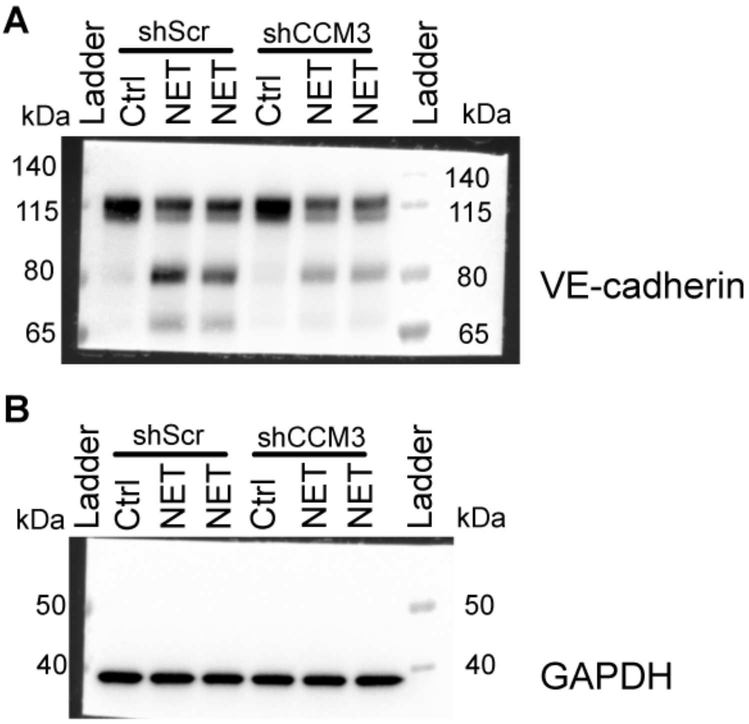

Supplementary Fig 1: Uncropped blots for Figures 3c (panel 1 and 4)
(A-B): Representative western blot images of VE-cadherin (A) and GAPDH (B) expression in HBMVECs (shscramble or shCCM3) stimulated with 500 ng/mL NET-enriched supernatant or control media for 24hrs.

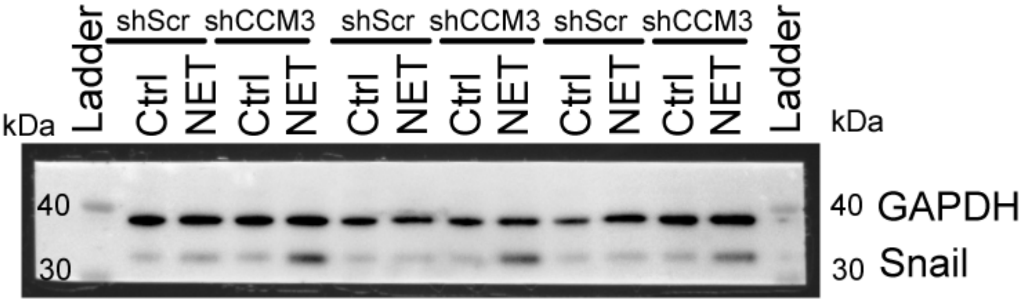

Supplementary Fig 2: Uncropped blots for Figures 3i (panel 1)
Representative western blot images of GAPDH (top band) and Snail (bottom band) expression in HBMVECs (shscramble or shCCM3) stimulated with 500 ng/mL NET-enriched supernatant or control media for 24hrs.

Source data file for Figure 1A:

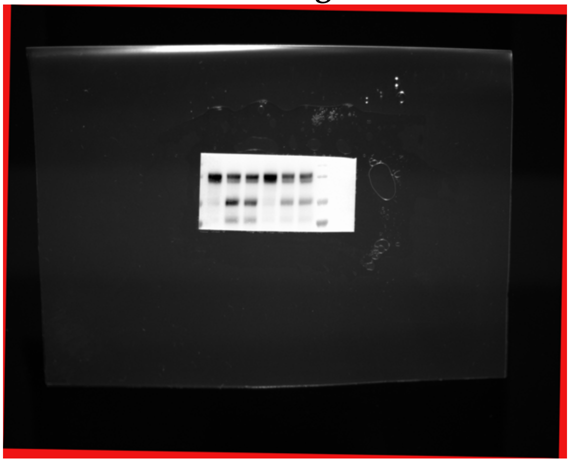

Source data file for Figure 1B:

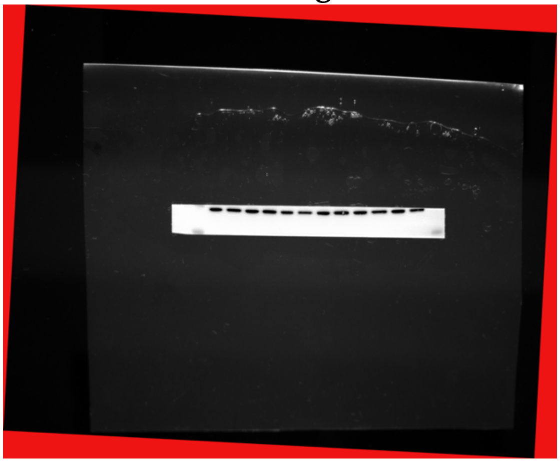

Source data file for Figure 2:

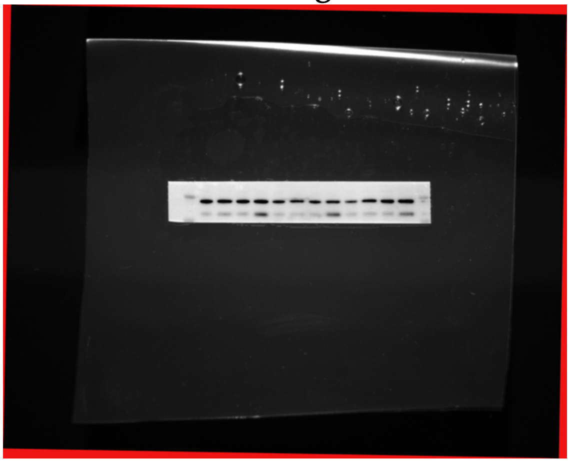

Supplement: Supplementary file 1 — Supplementary Figs. 1 and 2 containing full blots for Fig. 3c (panel 4) and Fig. 3i (panel 1). [file 44161_2024_577_MOESM1_ESM.pdf]
