# Supplementary material for: Pharmacological blocking of neutrophil extracellular traps attenuates immunothrombosis and neuroinflammation in cerebral cavernous malformation
Source: Nat Cardiovasc Res. 2024 Dec 4;3(12):1549–67. doi: 10.1038/s44161-024-00577-y (PMC11634782; doi:10.1038/s44161-024-00577-y)
Supplement: Supplementary file 2 — Reporting Summary [file 44161_2024_577_MOESM2_ESM.pdf]

nature portfolio

Corresponding author(s): Peetra Magnusson

Last updated by author(s): 22/10/2024

Reporting Summary

Nature Portfolio wishes to improve the reproducibility of the work that we publish. This form provides structure for consistency and transparency in reporting. For further information on Nature Portfolio policies, see our Editorial Policies and the Editorial Policy Checklist.

Statistics

For all statistical analyses, confirm that the following items are present in the figure legend, table legend, main text, or Methods section.

n/a	Confirmed	
☐	☒	The exact sample size (n) for each experimental group/condition, given as a discrete number and unit of measurement
☐	☒	A statement on whether measurements were taken from distinct samples or whether the same sample was measured repeatedly
☐	☒	The statistical test(s) used AND whether they are one- or two-sided<br>*Only common tests should be described solely by name; describe more complex techniques in the Methods section.*
☒	☐	A description of all covariates tested
☐	☒	A description of any assumptions or corrections, such as tests of normality and adjustment for multiple comparisons
☐	☒	A full description of the statistical parameters including central tendency (e.g. means) or other basic estimates (e.g. regression coefficient) AND variation (e.g. standard deviation) or associated estimates of uncertainty (e.g. confidence intervals)
☐	☒	For null hypothesis testing, the test statistic (e.g. F, t, r) with confidence intervals, effect sizes, degrees of freedom and P value noted<br>*Give P values as exact values whenever suitable.*
☒	☐	For Bayesian analysis, information on the choice of priors and Markov chain Monte Carlo settings
☒	☐	For hierarchical and complex designs, identification of the appropriate level for tests and full reporting of outcomes
☐	☒	Estimates of effect sizes (e.g. Cohen's d, Pearson's r), indicating how they were calculated

Our web collection on statistics for biologists contains articles on many of the points above.

Software and code

Policy information about availability of computer code

Data collection	Analysis of microscopy images was done with Fiji (Image J, Version 1.54f). Western blot images were quantified using the iBright Analysis Software (version 5.3.0).
Data analysis	Data analysis was carried out using GraphPad Prism (version 8.0.2). Custom built macros were used to make analysis semi-automated. These macros are available on Github (DOI: 10.5281/zenodo.10590829.)

For manuscripts utilizing custom algorithms or software that are central to the research but not yet described in published literature, software must be made available to editors and reviewers. We strongly encourage code deposition in a community repository (e.g. GitHub). See the Nature Portfolio guidelines for submitting code & software for further information.

Data

Policy information about availability of data

All manuscripts must include a data availability statement. This statement should provide the following information, where applicable:

- Accession codes, unique identifiers, or web links for publicly available datasets
- A description of any restrictions on data availability
- For clinical datasets or third party data, please ensure that the statement adheres to our policy

Bulk EC sequencing (GEO: GSE246373), single-cell RNA-sequencing (GEO: GSE155788; GSE98816, GSE99058 and GSE99235), and single-nucleus RNA-sequencing data GSE165371 data were retrieved from previously published studies.

Research involving human participants, their data, or biological material

Policy information about studies with human participants or human data. See also policy information about sex, gender (identity/presentation), and sexual orientation and race, ethnicity and racism.

Reporting on sex and gender	The patient cohort included ten patients with CCMs (male: female ratio 6:4) We did not observe any age or sex difference in the organized clots present in these patients.
Reporting on race, ethnicity, or other socially relevant groupings	No Reporting on race, ethnicity, or other socially relevant groupings was done.
Population characteristics	Validations of our findings on "organized clots" was done in human samples. The patient cohort included ten patients with CCMs (age 18-66 years; male: female ratio 6:4; sporadic: familial ratio 6:4).
Recruitment	Tissue samples were collected during routine surgical treatment of the patients for whom the decision for surgery had been done on clinical basis. For biopsies from Helsinki University Hospital, patients were invited to participate by their physicians. For biopsies from Allaince to cure cavernous malformations, patients were recruited via advertisements, or reached out directly to donate biopsy samples. In terms of bias, patients who donated biopsies are educated, and more likely females. This bias however does not impact the study's research findings.
Ethics oversight	The collection and use of the human samples in research was approved by organizational and ethical committees: Helsinki University Hospital (HUS/125/2018), the Committee on Research Ethics of Helsinki University hospital (HUS/3648/2017), and the Swedish Ethical Review Authority (EPM; 2019-04715, 2019-06374 and 2017-165).

Note that full information on the approval of the study protocol must also be provided in the manuscript.

Field-specific reporting

Please select the one below that is the best fit for your research. If you are not sure, read the appropriate sections before making your selection.

☒ Life sciences ☐ Behavioural & social sciences ☐ Ecological, evolutionary & environmental sciences

For a reference copy of the document with all sections, see nature.com/documents/nr-reporting-summary-flat.pdf

Life sciences study design

All studies must disclose on these points even when the disclosure is negative.

Sample size	37 mice were included in the study, a detailed data table on all mice used is presented as Supplementary Table 2. Sample size was determine d based on knowledge from previous drug treatments (Oldenburg at al, 2021), on knowledge on the variability in the model, and the proposed statistical method (a Mann whitney test requires n=4 per group)
Data exclusions	Mice (n=5) that died before the day of collection or had noticeable balance issues (n=1) were excluded from the study.
Replication	Results were derived from data of 13 biological replicates (vehicle) and 8 biological replicates (treated group); as well as averaged of 3 technical replicates (multiple litters collected at different times) were used. All attempts at replication were successful
Randomization	No statistical method used for randomization. For murine studies, mice genotypes were determines after which they were randomly assigned into treatment or vehicle groups. Allocation of cells to treatment or control conditions was done randomly
Blinding	The investigators were not blinded during the treatment because they had to calculate doses and inject the mice. Following collection, the mice were assigned unique IDs and the investigators blinded for subsequent data collection and analysis. For in vitro experiments, the investigators were blinded for data collection and analysis.

Reporting for specific materials, systems and methods

We require information from authors about some types of materials, experimental systems and methods used in many studies. Here, indicate whether each material, system or method listed is relevant to your study. If you are not sure if a list item applies to your research, read the appropriate section before selecting a response.

Materials & experimental systems

n/a	Involved in the study	
	☒	Antibodies
	☒	Eukaryotic cell lines
☒		Palaeontology and archaeology
	☒	Animals and other organisms
☒		Clinical data
☒		Dual use research of concern
☒		Plants

Methods

n/a	Involved in the study	
☒		ChIP-seq
☒		Flow cytometry
☒		MRI-based neuroimaging

Antibodies

Antibodies used

Alpha-SMA conjugated to Cy3 Mouse 1:500 Sigma C6198
Alpha-Tubulin Mouse 1:2000 Sigma-Aldrich T5168
Annexin A5 Rabbit 1:100 R&D AF1556
B-catenin Mouse 1:100 BD Transduction 610153
Carbonic anhydrase conjugated to AF488 Rabbit 1:100 Novus Bio NB100-417AF647
CCM3 Rabbit 1:200 Protein tech 10294-2-AP
CD13 Rat 1:100 Bio-Rad MCA2183
CD31 Goat 1:200 R&D AF3628
CD41 conjugated to PE Rat 1:200 BD Pharmigen 558040
CD42b Rabbit 1:100 Abcam ab183345
CD45 Goat 1:200 R&D AF114
CD93 Mouse 1:100 MBL D198-3
citH3 Rabbit 1:200 Abcam ab5103
Collagen IV Rabbit 1:100 BioRad 2150-1470
CX3CR1 conjugated to AF488 Mouse 1:50 Biolegend SA011F11
ERG Rabbit 1:100 abcam 196149
Fibrin/Fibrinogen Goat 1:500 Nordic MU Bic GAM/FbGt/75
GAPDH Rabbit 1:5000 Abcam ab181602
GFAP Rat 1:500 ThermoFisher B-0300
GLUT1 Rabbit 1:50 Abcam ab115730
Iba1 Rabbit 1:100 Abcam ab178847
ICAM-1 Goat 1:200 R&D AF 796
Isolectin B4 - 1:200 Vector labs B-1205
Ki67 Rabbit 1:100 Abcam ab16667
Ly6B 7/4 conjugated to 488 Rat 1:100 Abcam ab53453
Ly6G (IA8)-PE Rat 1:100 BD Pharmigen 551461
Myeloperoxidase Goat 1:200 R&D AF3667
N-cadherin Mouse 1:200 BD Biosciences 610920
Podocalyxn Goat 1:100-1:200 R&D AF1556
Sca1(Ly6a) Rat 1:100 Abcam ab51317
Snail (C15D3) Rabbit 1:1000 Cell signalling 3879
TER-119 Rat 1:500 BD Pharmingen 553671
Thrombomodulin Goat 1:100 R&D AF3894
VE-cadherin Rabbit 1:1000 Cell Signaling 2500
Vimentin Rabbit 1:100; 1:2000 (WB) Abcam ab92547
Vinculin (hVln) Mouse 1:500 Sigma Aldrich V9131
vWF Rabbit 1:500; 1:100 (cells) Dako A0082

Validation

All antibodies used were purchased from commercial vendors and were selected because they had been well validated by their manufacturers and in different publications for use in the species we used them for. Validation details and relevant publications are detailed on their respective websites

Eukaryotic cell lines

Policy information about cell lines and Sex and Gender in Research

Cell line source(s)

Primary human microvascular endothelial cells (HBMVECs) were purchased from iXCells (10HU-051) at passage 1 (p1). Lenti-X 293T cells were purchased from Takara (#632180) . sex undetermined

Authentication

Lenti-X 293T cells were authenticated by Takara. HBMVECs were authenticated by iXCells and by phenotypic characterization.

Mycoplasma contamination

All cell lines tested negative for mycoplasma contamination

Commonly misidentified lines (See ICLAC register)

No commonly misidentified cell line was used.

Animals and other research organisms

Policy information about studies involving animals; ARRIVE guidelines recommended for reporting animal research, and Sex and Gender in Research

Laboratory animals	C57BL/6J mice (Cdh5(PAC)-Cre-ERT2/Ccm3flox/flox) Ages: 14-28 days and C57BL/6J mice Cdh5(PAC)-Cre-ERT2/Ccm3flox/flox Cre-negative mice. Ages: 14-15 days were used in this study.<br>All the mice were housed in microisolator cages containing wood shavings and enrichment. They were kept in a climate-controlled environment with 12-h light/12-h dark cycles. Mice were fed standard rodent chow with free access to water and regularly monitored for health status.
Wild animals	No wild animals were used in this study
Reporting on sex	Both male and female mice were used for this study. There was no observed sex-specific effects.
Field-collected samples	No field collected samples were used in the study
Ethics oversight	All experiments involving animals were conducted according to the principles in the Swedish National Board for Laboratory Animals and European Convention for Animal Care. Animal experiments were approved by the regional ethics committees in Uppsala, Sweden (5.8.18-16224-2020).

Note that full information on the approval of the study protocol must also be provided in the manuscript.

Plants

Seed stocks	*Report on the source of all seed stocks or other plant material used. If applicable, state the seed stock centre and catalogue number. If plant specimens were collected from the field, describe the collection location, date and sampling procedures.*
Novel plant genotypes	*Describe the methods by which all novel plant genotypes were produced. This includes those generated by transgenic approaches, gene editing, chemical/radiation-based mutagenesis and hybridization. For transgenic lines, describe the transformation method, the number of independent lines analyzed and the generation upon which experiments were performed. For gene-edited lines, describe the editor used, the endogenous sequence targeted for editing, the targeting guide RNA sequence (if applicable) and how the editor was applied.*
Authentication	*Describe any authentication procedures for each seed stock used or novel genotype generated. Describe any experiments used to assess the effect of a mutation and, where applicable, how potential secondary effects (e.g. second site T-DNA insertions, mosiacism, off-target gene editing) were examined.*
