# Supplementary figures and images for: Pharmacological blocking of neutrophil extracellular traps attenuates immunothrombosis and neuroinflammation in cerebral cavernous malformation
Source: Nat Cardiovasc Res. 2024 Dec 4;3(12):1549–67. doi: 10.1038/s44161-024-00577-y (PMC11634782; doi:10.1038/s44161-024-00577-y)

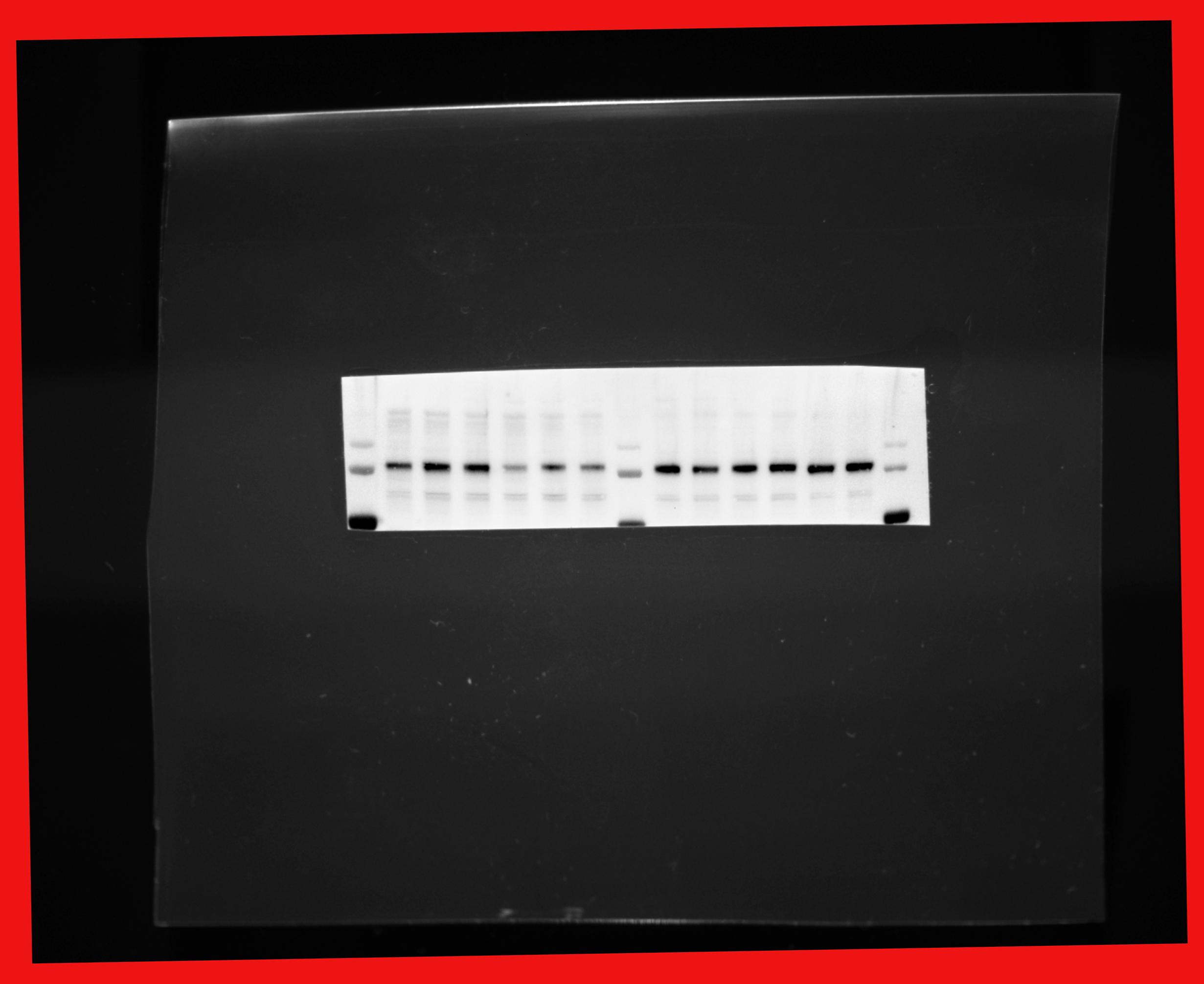

Supplement: Supplementary file 6 — A zip folder containing unprocessed western blots for Extended Data Fig. 3. [file 44161_2024_577_MOESM6_ESM.zip › Onyeogaziri_Western_blot_source_data/ED_Fig3Biii_Ncad.tif]

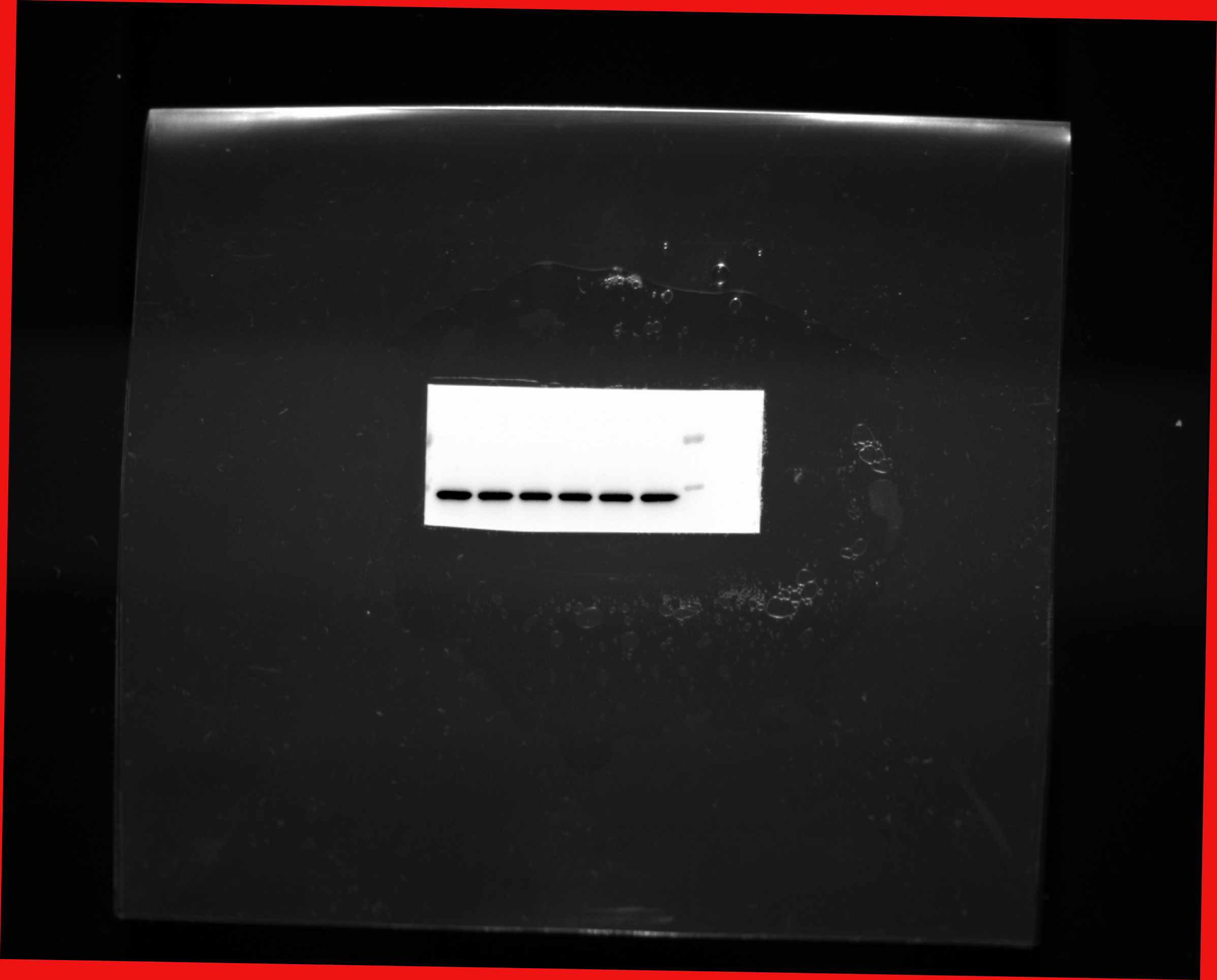

Supplement: Supplementary file 6 — A zip folder containing unprocessed western blots for Extended Data Fig. 3. [file 44161_2024_577_MOESM6_ESM.zip › Onyeogaziri_Western_blot_source_data/ED_Fig3Civ_GAPDH.tif]

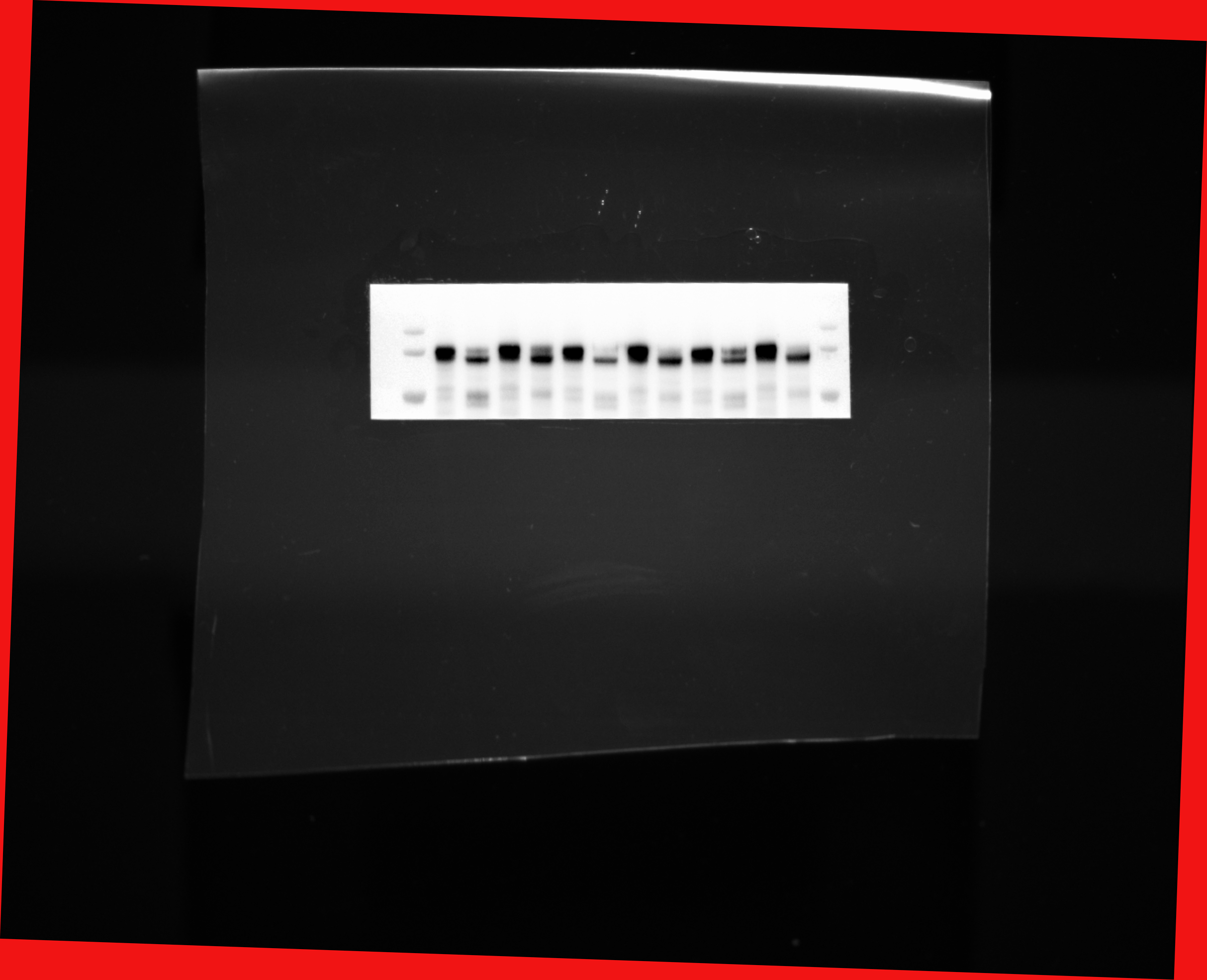

Supplement: Supplementary file 6 — A zip folder containing unprocessed western blots for Extended Data Fig. 3. [file 44161_2024_577_MOESM6_ESM.zip › Onyeogaziri_Western_blot_source_data/ED_Fig3Iiii_Vecad.tif]

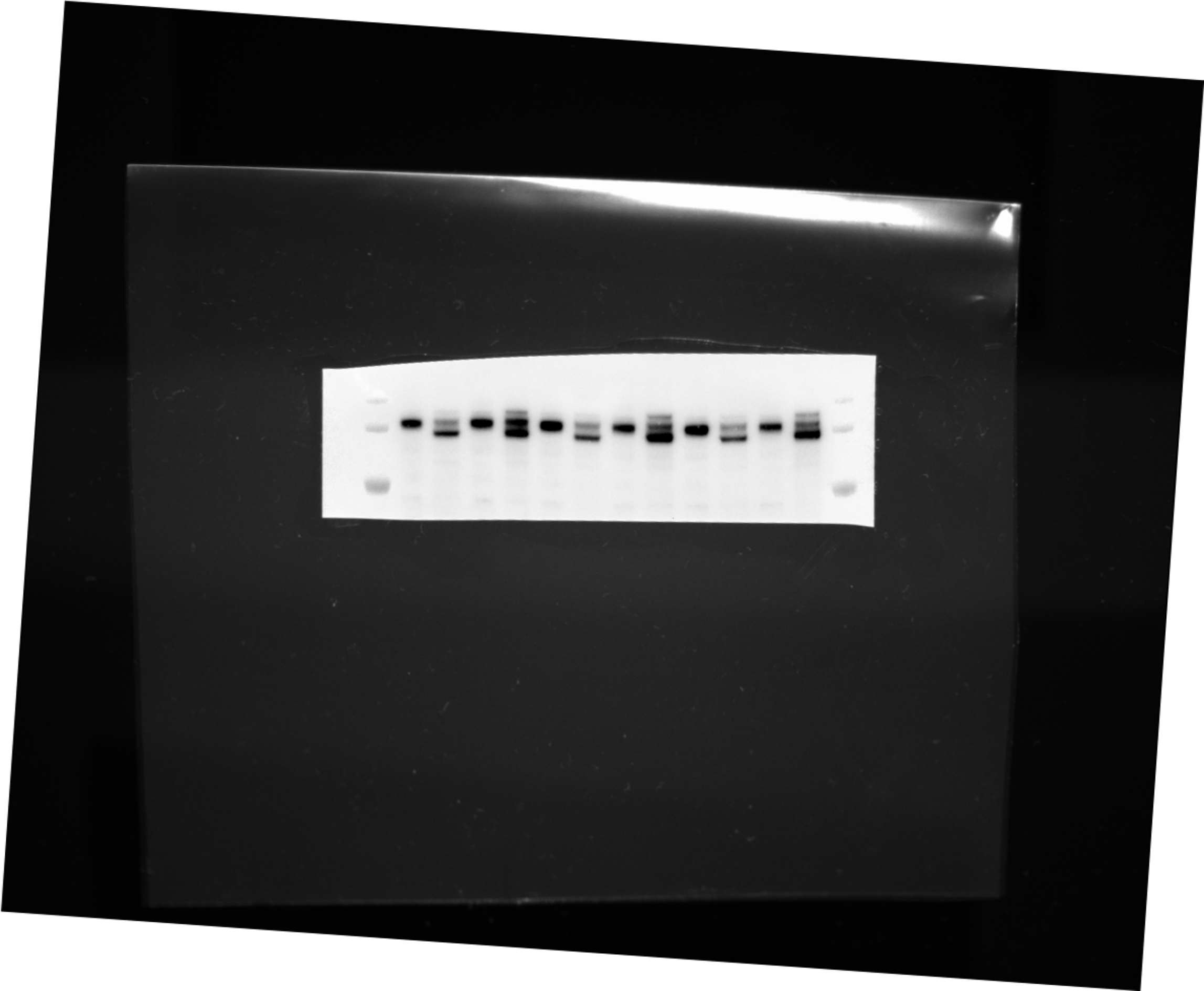

Supplement: Supplementary file 6 — A zip folder containing unprocessed western blots for Extended Data Fig. 3. [file 44161_2024_577_MOESM6_ESM.zip › Onyeogaziri_Western_blot_source_data/ED_Fig3Iii_Ncad.tif]

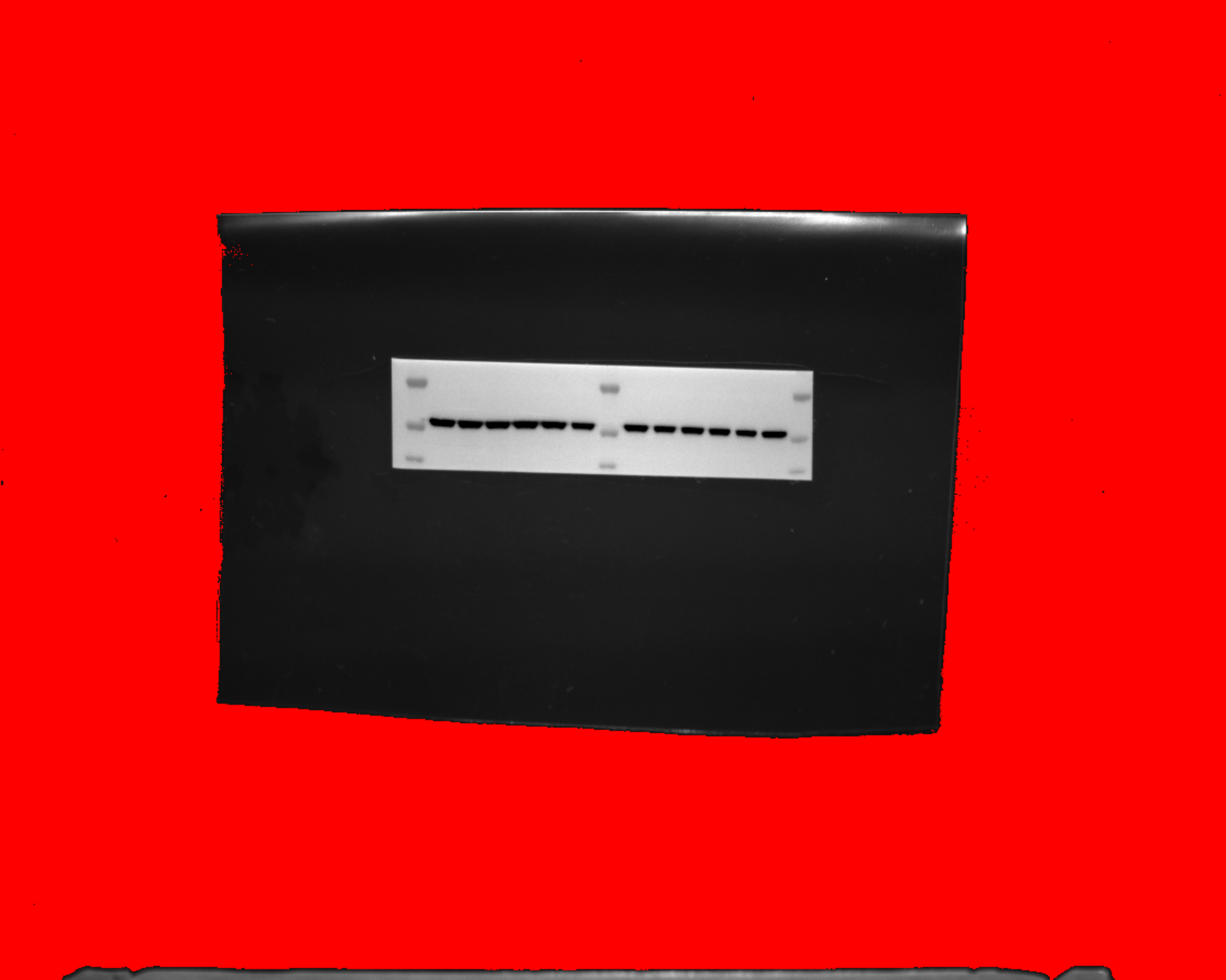

Supplement: Supplementary file 6 — A zip folder containing unprocessed western blots for Extended Data Fig. 3. [file 44161_2024_577_MOESM6_ESM.zip › Onyeogaziri_Western_blot_source_data/ED_Fig3Ciii_Tubulin.tif]

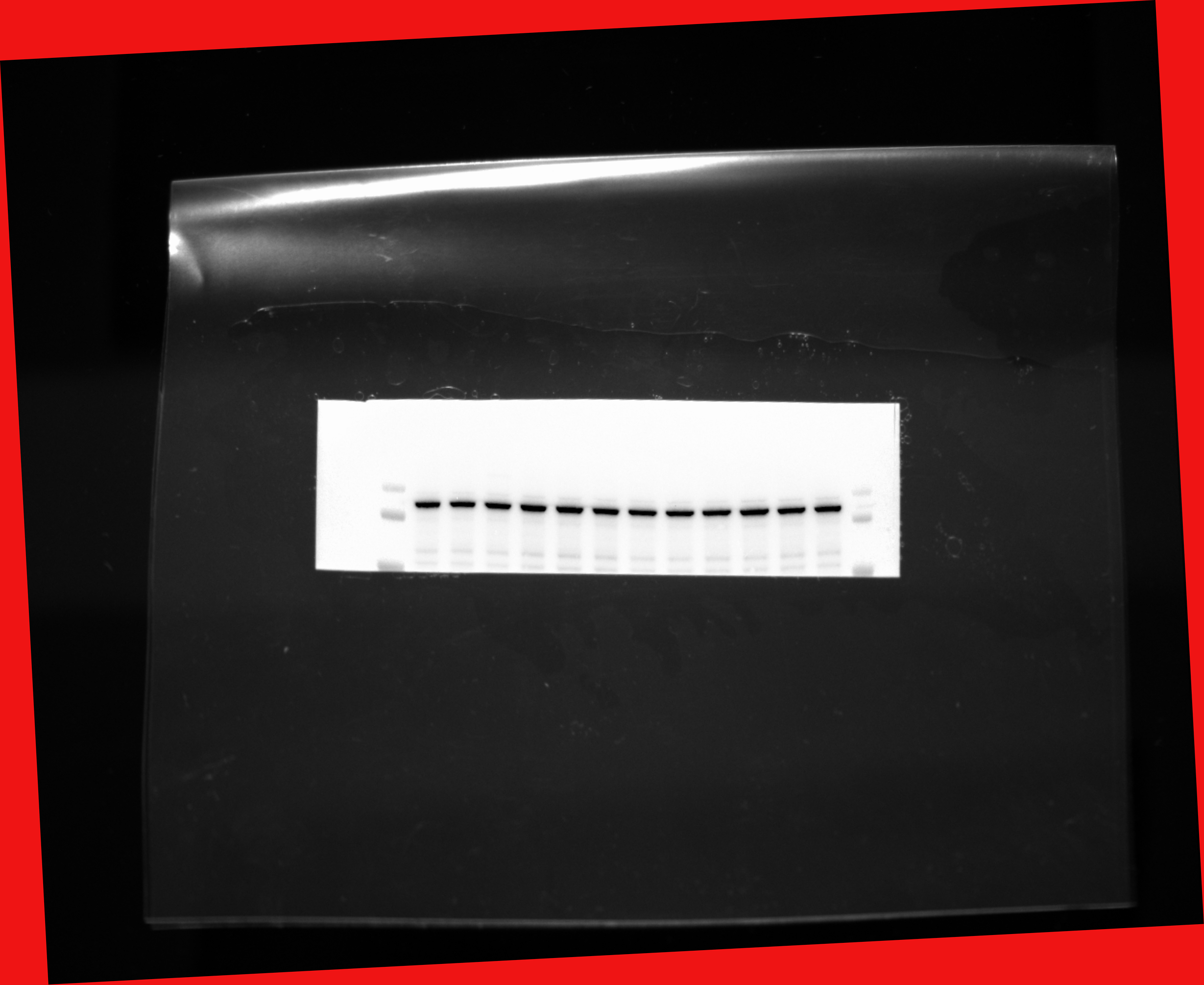

Supplement: Supplementary file 6 — A zip folder containing unprocessed western blots for Extended Data Fig. 3. [file 44161_2024_577_MOESM6_ESM.zip › Onyeogaziri_Western_blot_source_data/ED_Fig3Bii_Vinculin.tif]

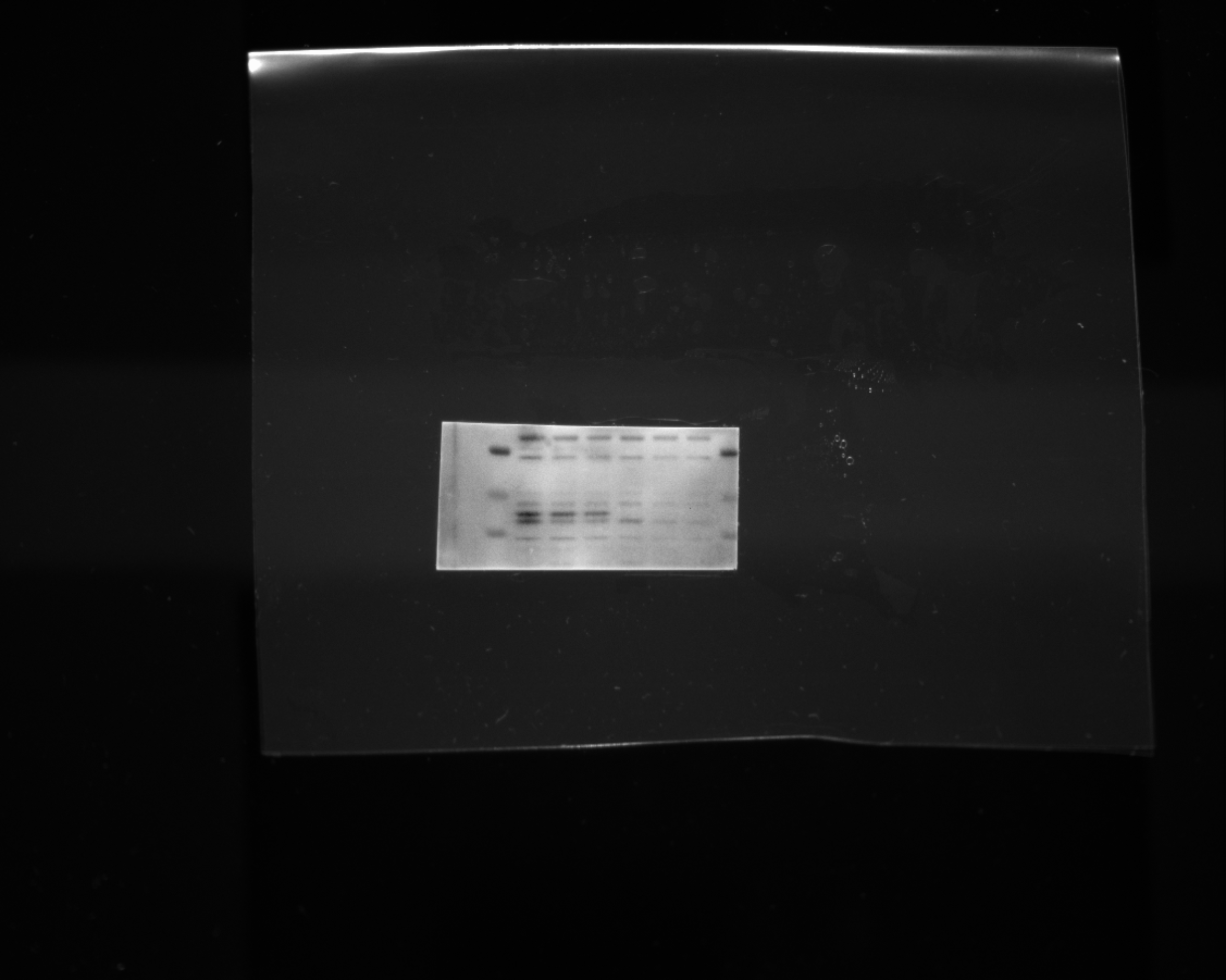

Supplement: Supplementary file 6 — A zip folder containing unprocessed western blots for Extended Data Fig. 3. [file 44161_2024_577_MOESM6_ESM.zip › Onyeogaziri_Western_blot_source_data/ED_Fig3Biv_CCM3.tif]

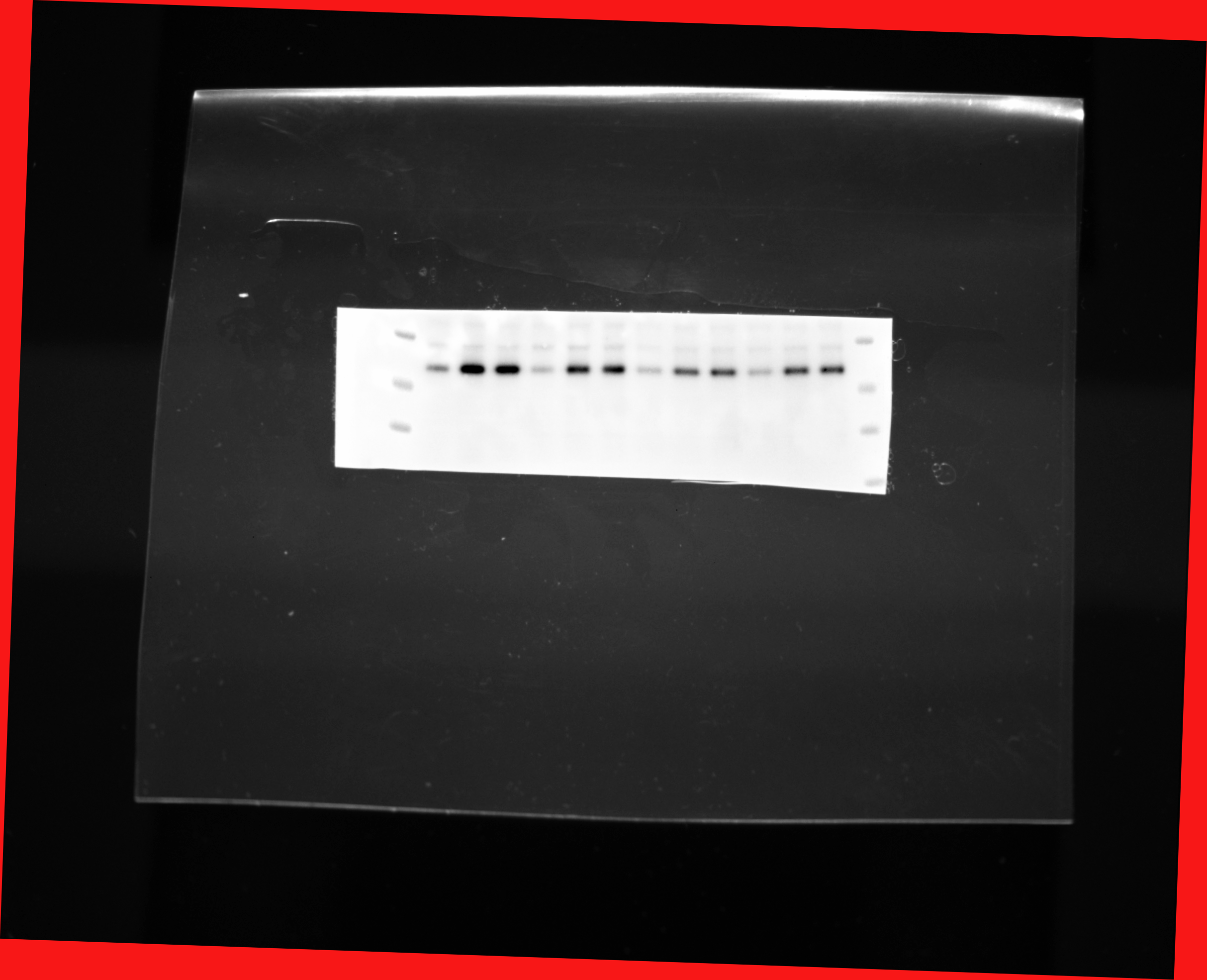

Supplement: Supplementary file 6 — A zip folder containing unprocessed western blots for Extended Data Fig. 3. [file 44161_2024_577_MOESM6_ESM.zip › Onyeogaziri_Western_blot_source_data/ED_Fig3Bi_Snail.tif]

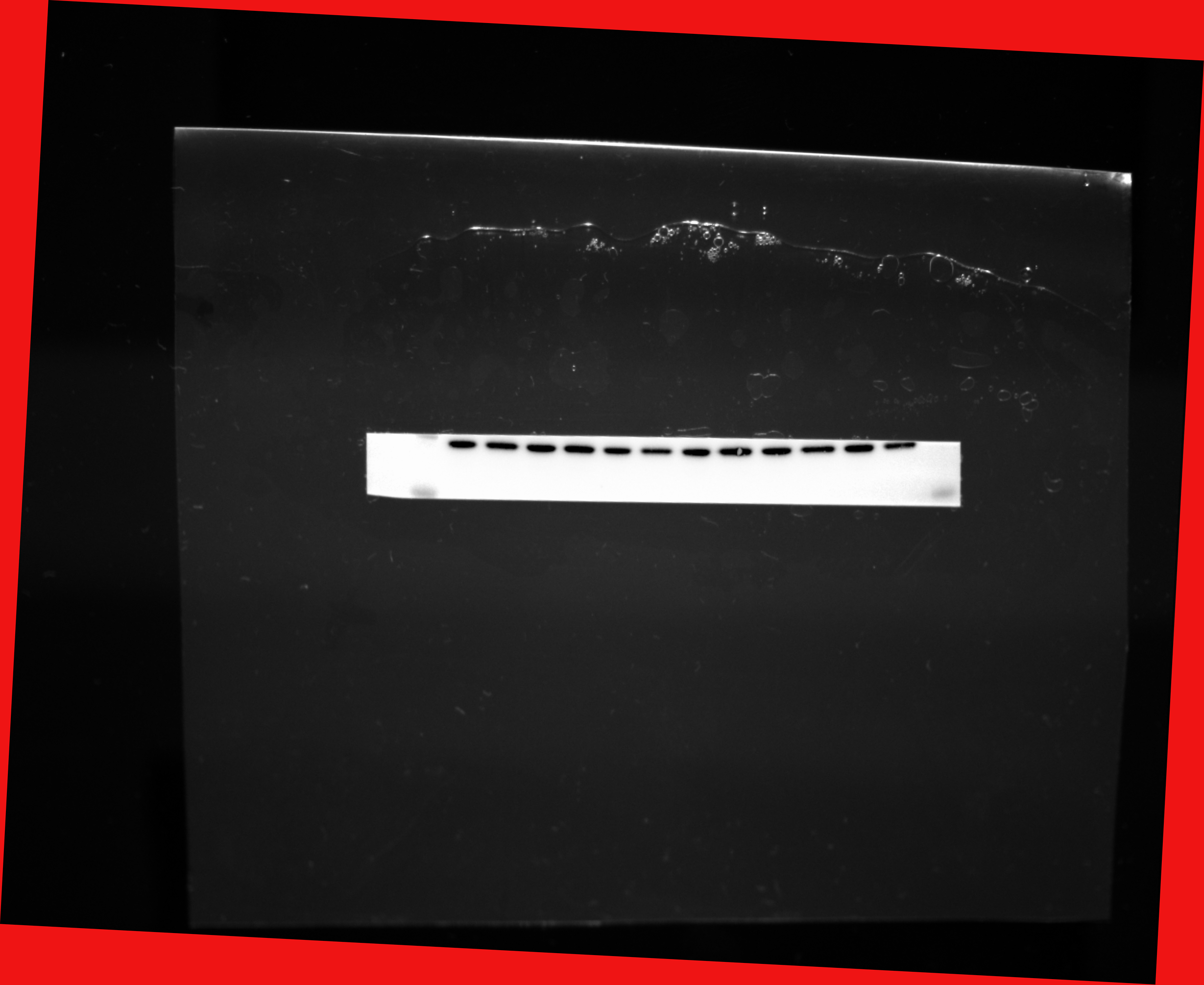

Supplement: Supplementary file 6 — A zip folder containing unprocessed western blots for Extended Data Fig. 3. [file 44161_2024_577_MOESM6_ESM.zip › Onyeogaziri_Western_blot_source_data/ED_Fig3Iv_GAPDH.tif]

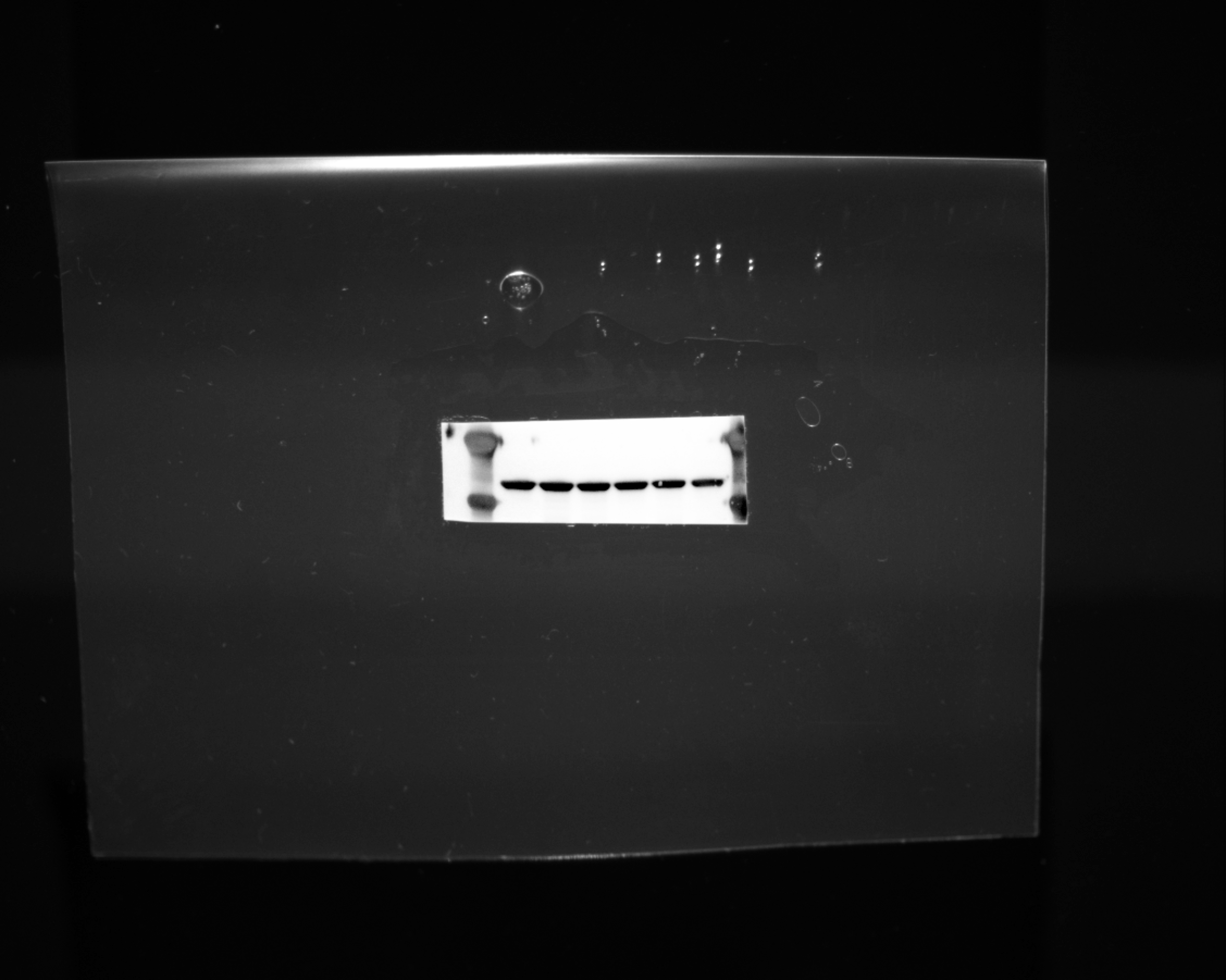

Supplement: Supplementary file 6 — A zip folder containing unprocessed western blots for Extended Data Fig. 3. [file 44161_2024_577_MOESM6_ESM.zip › Onyeogaziri_Western_blot_source_data/ED_Fig3Cii_Vimentin.tif]

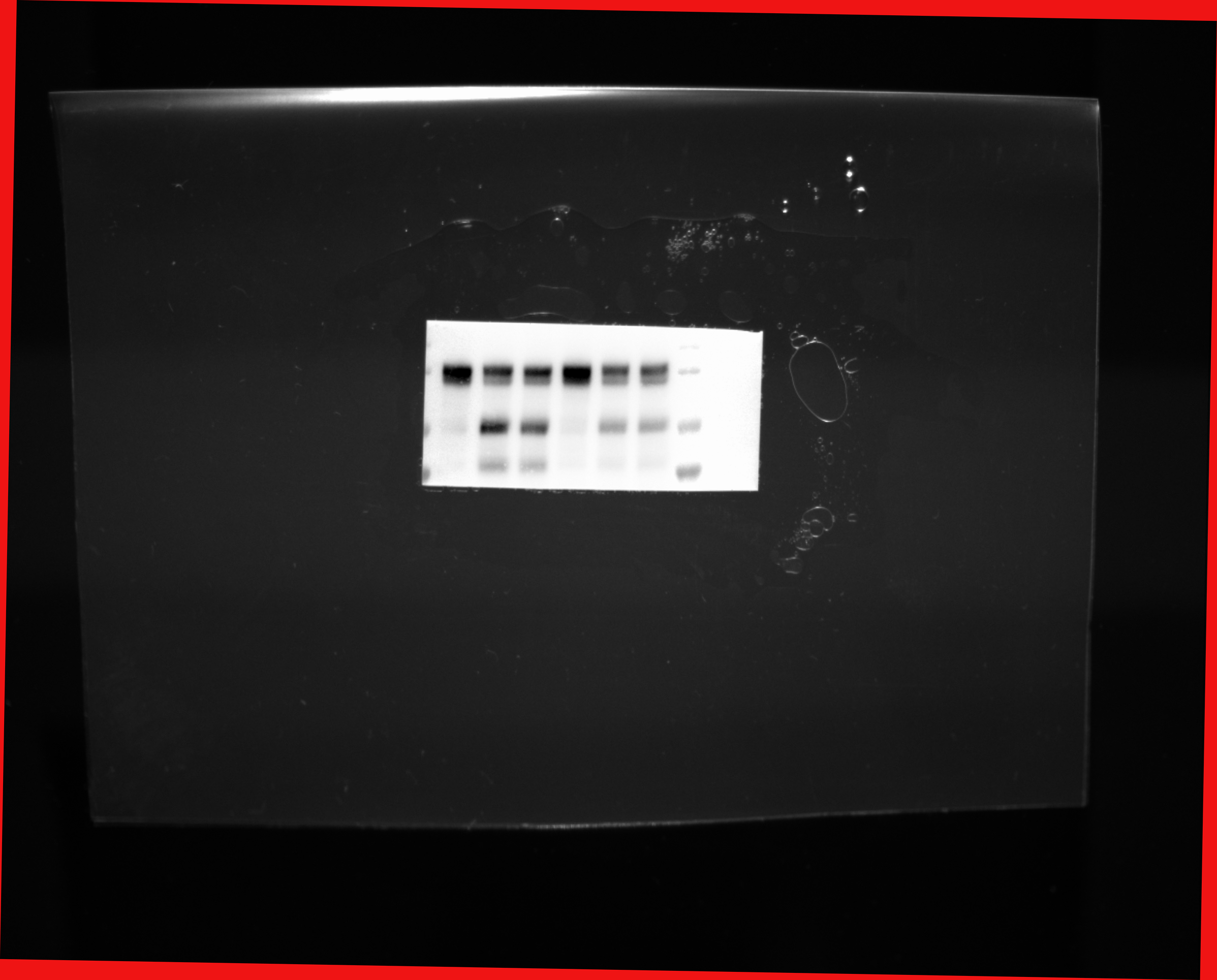

Supplement: Supplementary file 6 — A zip folder containing unprocessed western blots for Extended Data Fig. 3. [file 44161_2024_577_MOESM6_ESM.zip › Onyeogaziri_Western_blot_source_data/ED_Fig3Ci_Vecad.tif]

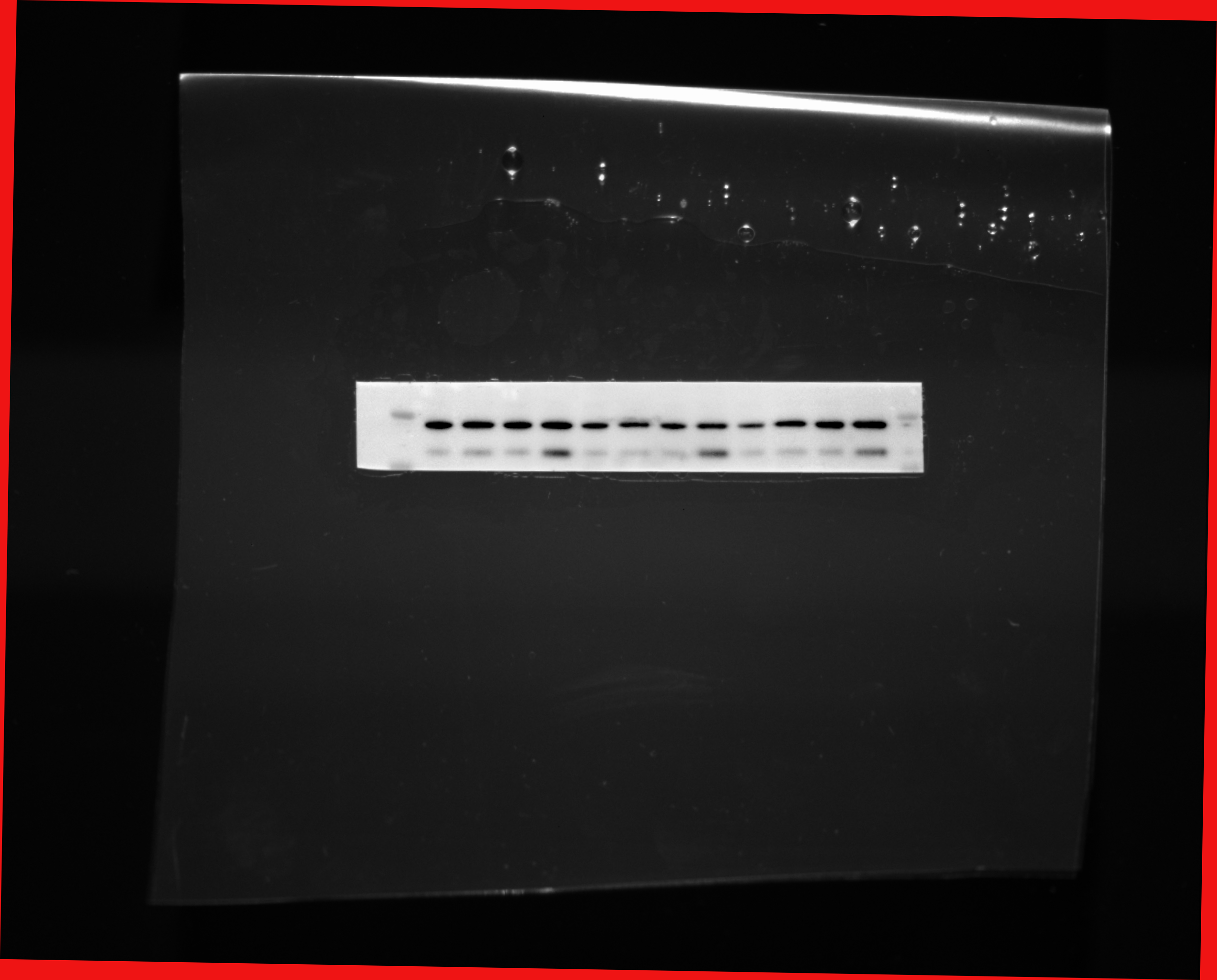

Supplement: Supplementary file 6 — A zip folder containing unprocessed western blots for Extended Data Fig. 3. [file 44161_2024_577_MOESM6_ESM.zip › Onyeogaziri_Western_blot_source_data/ED_Fig3Ii_Snail-lower band.tif]

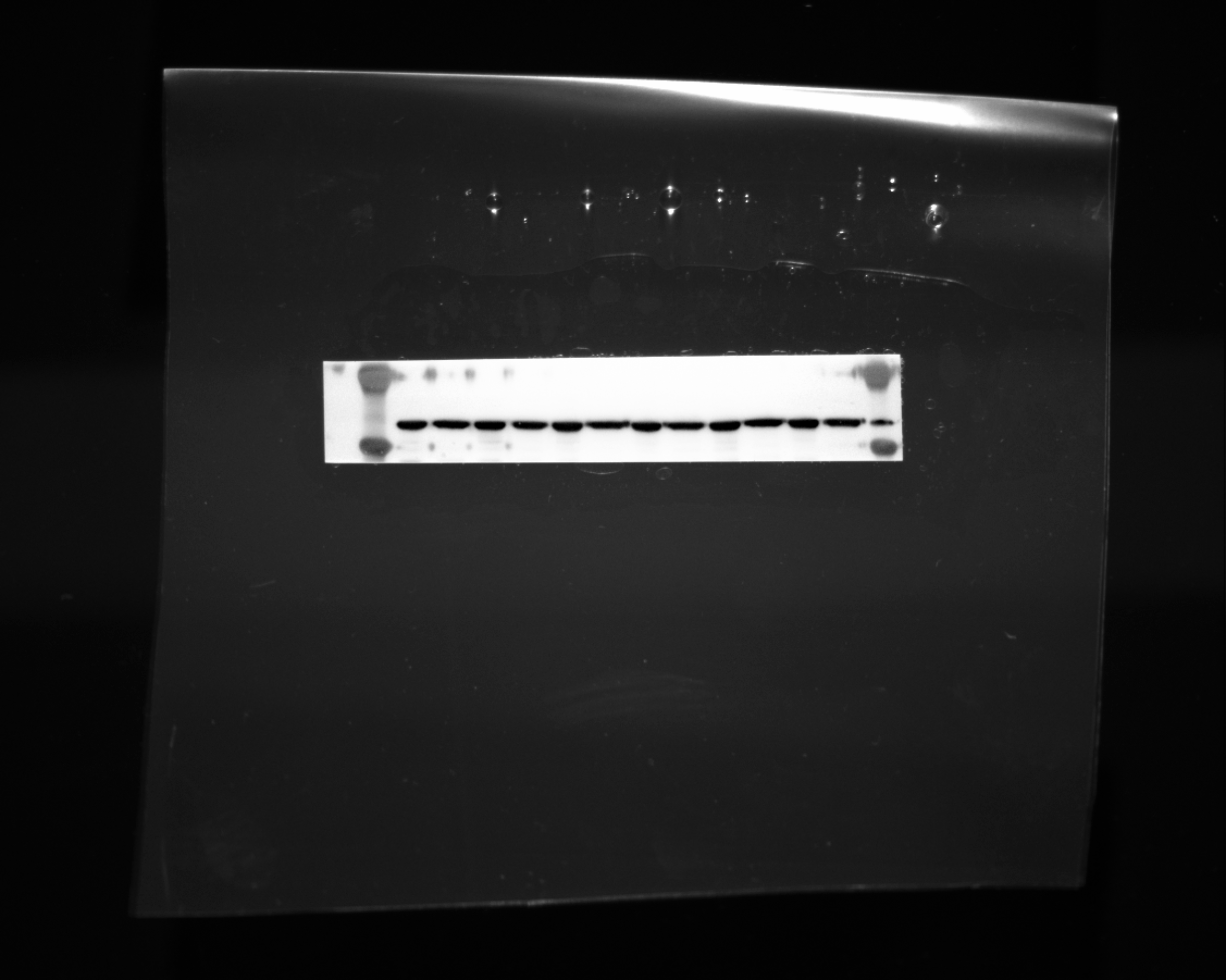

Supplement: Supplementary file 6 — A zip folder containing unprocessed western blots for Extended Data Fig. 3. [file 44161_2024_577_MOESM6_ESM.zip › Onyeogaziri_Western_blot_source_data/ED_Fig3Iiv_Vimentin.tif]
